# A dual transcriptome analysis reveals accession-specific resistance responses in *Lathyrus sativus* against *Erysiphe pisi*

**DOI:** 10.3389/fpls.2025.1542926

**Published:** 2025-03-05

**Authors:** Rita M. Maravilha, Telma Fernandes, Pedro M. Barros, Susana T. Leitão, Diego Rubiales, Maria Carlota Vaz Patto, Carmen Santos

**Affiliations:** ^1^ Genetics and Genomics of Plant Complex Traits, Instituto de Tecnologia Química e Biológica António Xavier, Universidade Nova de Lisboa, Oeiras, Portugal; ^2^ Resistlab, Instituto de Agricultura Sostenible, Consejo Superior de Investigaciones Científicas, Córdoba, Spain

**Keywords:** grass pea (*Lathyrus sativus* L.), dual-RNA sequencing, partial resistance, NLRs, *Erysiphe pisi* effectors

## Abstract

*Lathyrus sativus* (grass pea) is a valuable crop for sustainable agriculture, offering dietary benefits and desirable agronomic traits. However, its yield stability is limited by diseases such as powdery mildew caused by *Erysiphe pisi*. Increasing fungal resistance to pesticides and environmental concerns demand the development of resistant crop varieties. To identify key defense mechanisms and effector genes involved in the *Lathyrus sativus*-*Erysiphe pisi* interaction we analyzed four *L. sativus* accessions exhibiting varying resistance to *E. pisi* (resistant, partially resistant, partially susceptible, and susceptible) using a dual RNA-Seq experiment across different time points. We observed a host biphasic response, characterized by an initial burst of gene expression, followed by a quiescent phase, and a subsequent wave of intense gene expression. Common *L. sativus* defense mechanisms included antifungal protein expression, cell wall reinforcement, and reactive oxygen species-mediated defense. These defenses involved respectively Bowman-Birk type proteinase inhibitors, peptidyl-prolyl cis-trans isomerases and mannitol dehydrogenases. The resistant accession specifically activated early reinforcement of structural barriers associated with lignin biosynthesis and the phenylpropanoid pathway, along with sustained chemical defenses (e.g. *eugenol synthase 1*), epigenetic regulation, and oxidative stress responses thorough peroxidases and heat shock proteins. The partial resistant accession exhibited a front-loaded defense response at early infection stages. Contrastingly, the partially susceptible accession exhibited a weaker baseline defense, with a slower and less robust response targeting pathogen infection. We identified potential *E. pisi* effectors, including genes involved in cell wall hydrolysis (e.g. mannosidase DCW1), nutrient acquisition (e.g. secreted alpha-glucosidase), and virulence (e.g. SnodProt1), with a higher diversity of effectors identified in the susceptible accession. In conclusion, this study identifies novel targets such as NLRs and effectors, antifungal proteins and genes related to cell wall reinforcement, within the complex *Lathyrus sativus*-*Erysiphe pisi* interaction to support future breeding programs aimed at enhancing resistance to *E. pisi* in *L. sativus* and related species.

## 1 Introduction

*Lathyrus sativus* (grass pea) is an annual legume highly valued both as a nutritious food source for humans and as animal feed. As the most widely cultivated species within the *Lathyrus* genus, it combines significant dietary benefits with desirable agronomic traits (Peña-Chocarro and Peña, 1999; Lambein et al., 2019). *Lathyrus sativus* cultivation demands minimal inputs, demonstrating remarkable resilience, in challenging conditions such as drought, flooding, and poor soils (Rubiales et al., 2020; Gonçalves et al., 2022; Sanches et al., 2024). It is traditionally cultivated in drought-prone, marginal areas of South Asia and East Africa (Vaz Patto and Rubiales, 2014a). In Mediterranean regions, *L. sativus* is vital in supporting local economies (Gonçalves et al., 2022; Rubiales et al., 2020). Certain *L. sativus* accessions exhibit high resistance to air and soil-born fungal diseases (Vaz Patto et al., 2006; Vaz Patto and Rubiales, 2009, 2014b; Sampaio et al., 2021; Martins et al., 2022, 2023), making them valuable sources of resistance genes. Due to their phylogenetic proximity, *Lathyrus* spp. share many pathogens with *Pisum*, *Lens*, and *Vicia* genera that include crops like pea, lentil, and vetches.

Powdery mildew is one of the most widespread and damaging airborne fungal diseases (Sulima and Zhukov, 2022). In legumes, it is caused by obligate biotrophic ascomycetes from the order Erysiphales (Rubiales et al., 2015; Martins et al., 2020; Sulima and Zhukov, 2022). Powdery mildew on *Lathyrus* spp. is often caused by *Erysiphe pisi* (Vaz Patto et al., 2006; Martins et al., 2020), the same pathogen responsible for the disease in pea (*P. sativum*), which also infects species within the *Medicago*, *Vicia*, *Lupinus*, and *Lens* genera (Fondevilla and Rubiales, 2012). The management of powdery mildew in the field has traditionally relied on chemical fungicides. However, increasing resistance among fungal strains and increasing environmental concerns have pushed for alternative control strategies, including the breeding of disease-resistant varieties (Fondevilla et al., 2007; Mundt, 2014).

Plant disease resistance has been divided broadly into two categories: incomplete or partial resistance provided by quantitative disease resistance (QDR) genes and complete resistance mediated by resistance genes (R-genes) (Delplace et al., 2022). R-genes mediate the plant immune system, which has two layers. The first relies on pattern recognition receptors (PRRs) that detect pathogen-associated molecular patterns (PAMPs), activating PAMP-triggered immunity (PTI) (Zipfel and Robatzek, 2010; Roux et al., 2014; Lolle et al., 2020). However, pathogens can bypass this defense by secreting effectors that suppress PTI and hijack host proteins (Vleeshouwers and Oliver, 2014). In response, plants use intracellular resistance receptors such as nucleotide-binding leucine-rich domain proteins (NLRs) to recognize effectors, activating a stronger defense response called effector-triggered immunity (ETI) (Roux et al., 2014; Adachi et al., 2019; Li S. et al., 2020). This recognition leads to prolonged resistance through various immune response pathways like reactive oxygen species (ROS) production, hypersensitive response (HR), and systemic-acquired resistance (SAR) (Adachi et al., 2019; Li W. et al., 2020; Derevnina et al., 2021).

Research on powdery mildew resistance´s genetic and molecular bases has largely focused on Arabidopsis and important cereal crop species such as barley, and wheat (Yun et al., 2016; Zhang et al., 2016; Kuhn et al., 2017; Kusch and Panstruga, 2017; Hoseinzadeh et al., 2019). In contrast, the resistance mechanisms of legume crops, including *L. sativus*, remain underexplored, hampering the more efficient and effective development of disease-resistant varieties. Previous studies have identified three key genes linked to *E. pisi* resistance in pea: the recessive genes *er1* (also known as *MLO1*) and *er2*, and the dominant gene *Er3.* The HR plays a major role in pea resistance, which is governed by *er2* and *Er3* genes (Fondevilla and Rubiales, 2012; Barilli et al., 2014). In contrast, *er1* provides from complete to moderate levels of resistance by blocking fungus haustoria formation (Fondevilla et al., 2006; Fondevilla and Rubiales, 2012; Iglesias-García et al., 2015). However, concerns about the durability of these resistance genes, due to pathogen evolution, underscore the need for additional resistance sources (Fondevilla et al., 2013). Partial resistance is a potentially more durable approach than complete resistance, due to the reduced selective pressure imposed on the pathogen (McDonald and Linde, 2002; Niks and Rubiales, 2002). In the model legume *Medicago truncatula*, significant progress has been made in identifying quantitative trait loci (QTLs) for *E. pisi* disease symptoms (mycelium and conidia covering the leaf surface) (Ameline-Torregrosa et al., 2008; Yang et al., 2013). QTLs for resistance have been mapped to chromosomes 4 and 5, corresponding to the loci *Epp1* (on chromosome 4), *Epa1*, and *Epa2* (on chromosome 5), while a dominant resistance gene *MtREP1* (resistance to *Erysiphe pisi* race 1), was also identified and mapped on chromosome 5 (Ameline-Torregrosa et al., 2008; Yang et al., 2013). More recently, a dual RNA-Seq approach (Gupta et al., 2020) has provided deeper insights into the interactions between *M. truncatula* and *E. pisi*, revealing R-gene-mediated resistance involves transcriptional reprogramming, amplifying PTI signaling, activating the jasmonic acid/ethylene signaling network, and balancing growth-defense resource allocation. Susceptibility is linked to suppressed defense signaling, and reduced cell wall defenses (Gupta et al., 2020). Additionally, sugar transporters were found to mediate basal resistance to powdery mildew in *M. truncatula* (Gupta et al., 2021). In *Lathyrus* spp., a genome-wide association study (GWAS) identified 12 single nucleotide polymorphisms (SNPs) linked to disease severity to *E. pisi* in *L. sativus* mapped across all chromosomes, except in chromosome 1 (Martins et al., 2023). In *L. cicera*, three QTLs mapped on linkage groups I, II, and IV were associated with partial resistance to *E. pisi* (Santos et al., 2020). The characterization of the *MLO1* genes in both species revealed that *Lathyrus* MLO1 proteins belong to Clade V, a group linked with powdery mildew susceptibility in dicots (Santos et al., 2021).

Breeding for sustainable durable resistance in crops includes strategies like pyramiding major resistance genes and quantitative resistance genes. NLR proteins, central to ETI, display significant structural diversity and are typically classified into four classes based on their N-terminal domains: coiled-coil NLR (CC-NLR), Toll/interleukin-1 receptor NLR (TIR-NLR), G10-subclade CC NLR (CC_G10_-NLR), RESISTANCE TO POWDERY MILDEW 8-like CC NLR (CC_R_-NLR), and TIR-NB-ARC-like-β-propeller WD40/tetratricopeptide-like repeats (TNPs) (Kourelis et al., 2021). NLRs often function in networks, in which sensor NLRs recognize pathogen effectors, and other NLRs function as helpers that translate the effector recognition into HR (Adachi et al., 2019). For example, CC_R_-NLRs are often considered TIR-NLR helpers, while MADA-containing CC-NLRs are often considered helpers of other CC-NLRs (Adachi et al., 2019; Derevnina et al., 2021; Contreras et al., 2023).

*Erysiphe pisi* employs a sophisticated array of effector proteins to disrupt host cellular processes and establish fungal colonization. Recent studies have identified and characterized 7 and 167 (Gupta et al., 2020; Sharma et al., 2019, respectively) putative *E. pisi* effectors with many different functional annotations, which are integral to pathogenicity and host defense evasion (Bhosle et al., 2019; Sharma et al., 2019; Bhosle and Makandar, 2021). *Erysiphe pisi* effector expression varies according to the infection stage and the specific host (Gupta et al., 2020). Among the identified *E. pisi* effector candidates there are Egh16H homologues, ribotoxins/ribonucleases, glycoside hydrolases, and heat shock proteins (Sharma et al., 2019; Gupta et al., 2020). Bhosle et al. (2019) uncovered that the *er2* resistance gene in *P. sativum* accession JI-2480 suppressed three different *E. pisi* effectors, highlighting the dynamic interplay between *E. pisi* effectors and host resistance mechanisms.

This work aimed to explore the transcriptomic networks involved in the interaction between *L. sativus* and *E. pisi* in resistant, partially resistant, and partially susceptible accessions compared to a susceptible accession. We used dual RNA-Seq to reveal the key host resistance-related genes (including NLRs), and pathogen effectors influencing this interaction, to deepen our understanding of the resistance mechanisms in *L. sativus* and the virulence strategies of *E. pisi*. This knowledge could significantly contribute to breeding programs in *L. sativus* and other legumes susceptible to *E. pisi*, such as pea.

## 2 Materials and methods

### 2.1 RNA-plant material, growing conditions and pathogen inoculation

Four contrasting *L. sativus* accessions were selected from an *L. sativus* worldwide collection previously phenotyped for the response against *E. pisi* using detached leaflets under controlled conditions (Martins et al., 2023): PI268478, PI221467_A, PI426882, and PI426890, rated as resistant (R), partially resistant (PR), partially susceptible (PS), and susceptible (S), respectively. Seedlings were grown in 0.5 L pots containing 250 cm^3^ of peat in a growth chamber at 22/20°C, 12-h light/12-h dark photoperiod. Fourteen-day-old whole plant seedlings were inoculated with *E. pisi* isolate Ep-CO-01. This *E. pisi* isolate was maintained on seedlings of the susceptible pea cv. ‘Messire’, at the Institute for Sustainable Agriculture – CSIC (Cordoba, Spain). Inoculation was performed on entire plants (undetached leaves) with *E. pisi* spores in five independent inoculation events corresponding to the five different inoculated time points: 6, 12, 24, 48, 72 hours after inoculation (hai). Each inoculation was done onto three plants (biological replicates) per accession, except for PR at 48 hai (1 biological replicate, due to insufficient plant material). Additionally, two cv. ‘Messire’ pea plants were used as inoculation control. For the inoculations, a settling tower was used to ensure uniform conidial deposition of 8 conidia/mm^2^ onto each seedling. After inoculation, the entire plants were kept in a growth chamber at 24/22°C, under a 12-h light/12-h dark photoperiod. Leaflets from each plant were sampled at 0 (non-inoculated), 6, 12, 24, 48, and 72 hai and were immediately frozen in liquid nitrogen and stored at -80°C until RNA isolation. Time points 0, 12, 48, and 72 hai were selected for RNA-Seq analysis based on histological evidence on the different infection stages of *E. pisi* in the closest species *P. sativum* (Barilli et al., 2014). Therefore, we selected 0 hai as a baseline before pathogen interaction; 12 hai to capture early infection events; 48 hai to represent fungal establishment; and 72 hai as a later infection stage, where differences between accessions become more pronounced. The experimental design is depicted in **Supplementary Figure S1**. Seven and fourteen days after inoculation, disease severity (DS) and infection type (IT) were visually estimated on the leaflets of *L. sativus* accessions and the pea cv. ‘Messire’ susceptibility control (**Supplementary Figure S2**). DS was scored as the percentage of leaflet area covered by mycelia. IT was recorded according to a 0 to 4 scale, where 0 corresponds to no visible disease symptoms, 1 = brown necrotic lesions with little or no mycelial development, 2 = some necrosis and chlorosis with slight to moderate mycelial development, 3 = moderate mycelial development with little chlorosis, and 4 = well-developed, freely sporulating colonies, with no necrosis or chlorosis (Trabanco et al., 2012).

### 2.2 RNA isolation, library construction and sequencing

For total RNA isolation, frozen leaflets were ground to a fine powder in liquid nitrogen using a mortar and pestle, and RNA was isolated using the GeneJET™ Plant RNA Purification Mini Kit (Thermo Scientific™, Massachusetts, USA) according to the manufacturer’s instructions. RNA integrity and DNA contamination were assessed by electrophoresis in a 1.2% agarose gel stained with SYBR™ Safe (Life Technologies™, California, USA). Trace amounts of DNA contamination were removed from RNA with treatment with TURBO™ DNase (Invitrogen™ by Thermo Fisher Scientific™, California, USA), following the manufacturer’s instructions. RNA concentration was measured using a Qubit 2.0 fluorometer with the Qubit RNA BR (Broad-Range) Assay Kit (Life Technologies™, California, USA). RNA purity was estimated based on the 260/280 and 260/230 absorbance ratios using a NanoDrop™ 2000c Spectrophotometer (Thermo Scientific™, Passau, Germany). The RNA library construction was carried out using a Stranded mRNA Library Preparation Kit (Roche/KAPA mRNA HyperPrep kit) and samples were sequenced using Illumina Novaseq PE150 (paired-end 150 bp) at STABvida sequencing provider (Lisbon, Portugal).

### 2.3 Bioinformatics analysis

For data analysis, all the reads from the sequencing data were subjected to a quality check using FastQC v0.11.9 (Andrews, 2010). Adaptor, barcodes, and low-quality reads (Phred score < 20) were removed using Cutadapt v4.0 (Martin, 2011) and high-quality reads were aligned to the *L. sativus* genome (JIC_Lsat_v2.1.1) (Vigouroux et al., 2024) using HISAT2 v2.2.1 with paired-end parameters (Kim et al., 2015) (**Supplementary Table S1**). Read counts per gene were obtained using featureCounts v2.0.6 (Su et al., 2014) based on the corresponding genome annotation. To analyze the relationship among biological replicates and the differences among time points and accessions, a principal component analysis (PCA) was done using the normalized gene counts per sample. Differential expression analysis was performed using the DESeq2 R package by comparing the expression profile of the PS, PR, or R accessions to the S accession, for each time point. False discovery rate (adjusted *P*-value) < 0.05 and |log_2_ fold change| > 1.0 were set as the thresholds for significant differential expression (**Supplementary Table S2**).

Clustering analysis of differentially expressed genes (DEGs) based on expression patterns was performed using k-means clustering. To determine the optimal number of clusters, we applied hierarchical clustering with Ward’s method and visually inspected the dendrogram. Due to the limited functional annotation of the JIC_Lsat_v2.1.1 proteome, Mercator4 v6.0 (Lohse et al., 2014) was used to predict the functional annotation of each DEG identified. To increase the protein annotation rate, two additional tools were selected within Mercator: the annotation tool ProtScriber v0.1.3 and the BLAST tool, which provides Swiss-Prot protein annotations for similar proteins using the Swiss-Prot dataset of Viridiplantae proteins. To complement the gene functional annotation of DEGs, candidate *A. thaliana* orthologues were identified using Blastp (v2.15) analysis based on *L. sativus* predicted protein sequences (Vigouroux et al., 2024). Only the best hits with an *e-*value < 0.001 were selected. Gene Ontology (GO) functional enrichment analysis of the DEGs was performed with g:Profiler (Kolberg et al., 2023) with Benjamini–Hochberg multiple testing correction (*P-*value< 0.05) (Benjamini and Hochberg, 1995).

NLRtracker v1.3.1 (Kourelis et al., 2021) was used to predict complete nucleotide-binding leucine-rich repeats (NLRs) on the JIC_Lsat_v2.1.1 proteome. To accurately assign each NLR to a class, the RefPlantNLR dataset (Kourelis et al., 2021) was aligned together with the NB-ARC output file of NLRtracker using Clustal Omega v1.2.2 within Geneious Prime 2022.2.2 (Sievers and Higgins, 2014). An NLR phylogenetic tree (**Supplementary Figure S3**) was obtained using the FastTree algorithm that, with the pseudo-counts setting, generated local bootstrap values based on 1000 resamples (Price et al., 2010). The tree was rooted using non-plant NLRs from the RefPlantNLR dataset (Kourelis et al., 2021). The clades for each NLR class were identified based on phylogenetic clustering with the respective reference NLRs.

Heat maps were generated using the heatmaply R package. The expression values were an average of three biological replicates, except for the PR accession at 48 hai, which only pertained to a single biological replicate.

For *E. pisi* candidate effector prediction, the high-quality RNA-Seq reads from inoculated samples (12, 48 and 72 hai) were aligned to the *E. pisi* ASM20880v1 NCBI genome using HISAT2 2.2.1 (Kim et al., 2015). Uniquely mapped aligned reads (*E. pisi* specific) were extracted using samtools v1.9 (Li et al., 2009) and the merged left and right reads were used to build a *de novo* transcriptome using Trinity (Grabherr et al., 2011). This transcriptome was then used as a reference to estimate gene expression by aligning again the RNA-Seq reads using Salmon v1.10.2 (Patro et al., 2017). *E. pisi* transcript counts were normalized for transcripts per million, accounting for the differences in total number of fungal reads between accessions and time points.

*Erysiphe pisi* effector candidates were predicted in the *de novo* transcriptome open reading frames using Predector (Jones et al., 2021) applying a minimum Predector score threshold of 0. Effector protein structures were subsequently predicted using ColabFold (Mirdita et al., 2022). Only structures with a predicted template modeling score (TM-score) greater than 0.5 were retained for further analysis. Foldseek (van Kempen et al., 2023) was used to search for effectors with structural similarities to known effector structures. The predicted structures were compared to a Foldseek database (https://zenodo.org/records/6480453), with 26,675 known effector structures of 21 species (Seong and Krasileva, 2023). On the other hand, the BLASTp tool was used against the NCBI fungi database for sequence-based effector functional prediction, using an *e-*value < 1E-0.5.

### 2.4 RNA-seq validation using quantitative real-time PCR

To validate RNA-Seq data and follow the whole infection process (0, 6, 12, 24, 48, and 72 hai) nine DEGs were selected: *inactive beta-amylase 9* (g5179.t1), *Kunitz trypsin inhibitor 5* (g3907.t1), *NAC domain-containing protein JA2L* (g1304.t1), *peptidyl-prolyl cis-trans isomerase FKBP65* (g28159.t1), *eugenol synthase 1* (g18115.t1), *pathogenesis-related protein 10* (g4535.t1), *adagio protein 3* (g27034.t1), *protein EARLY FLOWERING 4* (g19671.t1), *glycine-rich RNA-binding protein 7* (g29906.t1). These genes were selected based on their high expression variation on the different accessions and time points. cDNA was synthesized from 1 μg of total RNA from each sample following the manufacturer’s instructions of the iScript™ cDNA synthesis kit (Biorad, California, USA). For RNA-Seq validation by RT-qPCR, the comparisons were performed using the 0 hai time point (non-inoculated) as the reference to the corresponding accession.

Specific primers were designed using the Primer3Plus online tool (https://primer3plus.com/) (Boston, USA), and checked for specificity using the Primer-BLAST tool (NCBI, USA). Primers were designed in the 3′ intra-exonic regions and were synthesized by STABvida (Caparica, Portugal) (**Supplementary Table S3**).

Four reference genes previously identified in *Lathyrus* species were selected to evaluate expression stability in the samples and conditions under study: *β-tubulin* (contig nr a6507;507), *γ-tubulin* (contig nr a77720;50), *histone H2A.2* (contig nr a20510;122), and *chromodomain helicase DNA-binding protein* (contig nr a1310;251) (Almeida et al., 2015; Santos et al., 2018). The expression stability was tested using the geNorm (Vandesompele et al., 2002), NormFind (Andersen et al., 2004), BestKeeper (Pfaffl et al., 2004), the ΔCt method (Silver et al., 2006), and RefFinder (Xie et al., 2012) online tool (https://blooge.cn/RefFinder/).

The relative expression of the nine selected target genes was determined by quantitative real-time PCR (RT-qPCR). The RT-qPCR reactions were performed using three biological replicates per accession (S, PS, PR, R) and six time points (0, 6, 12, 24, 48, and 72 hai). RT-qPCR was performed in a final volume of 20 µl, containing 0.5 ng of cDNA, 0.5 µM of each primer (except for pathogenesis-related protein 10 where 1 µM was used), and 1 x LightCycler^®^ 480 SYBR Green I Master. Thermal cycling for target and reference genes started with a denaturation step at 95°C for 5 min, followed by 40 cycles of denaturation at 95°C for 10 s and 60°C for 30 s. At the end of all gene expression cycling protocols, melting curve analysis was performed to validate amplification specificity under the following conditions: 65°C for 1 min to 97°C with the increment of 0.5°C for 11 s. In addition, a negative template control (NTC) without cDNA was included in each PCR plate to detect possible genomic DNA contaminations. The relative expression values (fold change-FC) of the nine target genes were normalized to the non-inoculated samples (0 hai) and the two reference genes showing the highest expression stability using the Pfaffl method (-Efficiency ^ΔΔCt^) (Pfaffl, 2001). Finally, FC data were transformed into a logarithmic scale (base 2) for graphical representation and statistical analyzes. ANOVA, followed by Dunnett’s multiple comparisons test, was performed to compare the expression levels of each time point to the non-inoculated sample per accession. Linear regression was performed to assess the relationship, and Pearson’s correlation test was used to evaluate the correlation between the log_2_ FC values of RNA-Seq and RT-qPCR. The data was analyzed using R statistical software version 4.3.0 (R Core Team, 2022) and GraphPad Prism 6 (GraphPad Software Inc.; San Diego, CA, USA).

## 3 Results

### 3.1 Dual RNA-seq analysis and RNA-seq validation by RT-qPCR

The selected *L. sativus* accessions (S, PS, PR, R) presented different whole plant disease severities (DS) at 7 and 14 days after inoculation (dai), as reported for detached leaflet assays (Martins et al., 2023) (**Supplementary Figure S2**). Using the Trabanco et al. (2012) disease symptoms visual scale, we observed a moderate mycelial development without sporulation or necrosis for all accessions at 7 dai, and for R and PR accessions at 14 dai (IT=3) (**Supplementary Figure S2**). At 14 dai, we observed abundant mycelial development and profuse sporulation for S and PS (IT=4). By 14 dai, DS had increased for all accessions, although macroscopic sporulation was more evident in S and PS than in PR and R, with S and PS reaching similar DS to pea cv. ‘Messire’ at 7 dai (**Supplementary Figure S2**).

**Figure 1:**
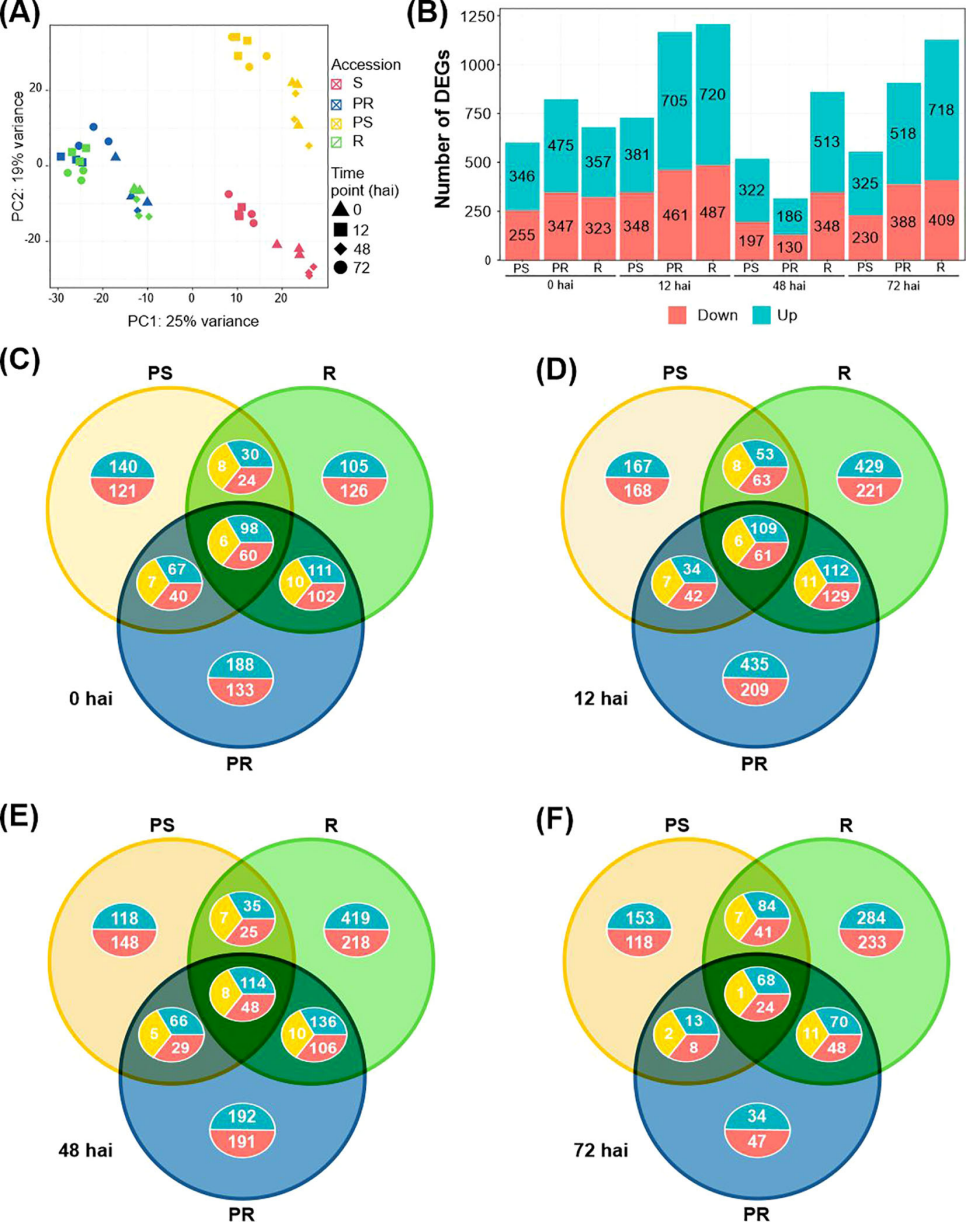
Overview of transcriptome data and differentially expressed genes (DEGs) in the *Lathyrus sativus* response to *Erysiphe pisi*. **(A)** Principal component analysis of the time-series transcriptomes of the four contrasting accessions to *E. pisi* based on counts per million values of all genes. **(B)** Number of DEGs upregulated (blue) and downregulated (pink) for comparisons under study. **(C-F)** Venn diagrams showing unique and common DEGs between *L. sativus* accessions for each time point: 0 hai **(C)**, 12 hai **(D)**, 48 hai **(E)** and 72 hai **(F)**. R, resistant; PR, partially resistant; PS, partially susceptible. Red: downregulated genes; blue: upregulated genes; yellow: contrasting gene expression.

The gene expression PCA of the contrasting *L. sativus* accessions to *E. pisi* showed that the three biological replicates at each time point clustered closely (**Figure 1A**). Furthermore, *L. sativus* samples revealed distinct gene expression patterns based primarily on accession (**Figure 1A**). Looking at the first two principal components, both S and PS samples clustered apart from the rest of the samples, separating from R and PR on PC1 and distinguishing between themselves on PC2. On the other hand, R and PR accessions clustered together with slight differences mostly on PC2, showing a more similar transcriptional response to *E. pisi* infection. When focusing on the separation per time point within each accession, there were roughly two groups: samples from 0 hai and 48 hai clustered closer together in all accessions, while samples at 12 and 72 hai formed a separate group.

We identified a total of 3,109 DEGs by comparing the expression profile of the PS, PR, or R accessions to the S accession, for each time point. All comparisons revealed more upregulated genes than downregulated genes across the study (**Figure 1B**). DEGs commonly identified at 0 hai (non-inoculated conditions) and at least one inoculated comparison were selected to represent the basal defense response of *L. sativus* to *E. pisi*. The largest number of DEGs was identified for the R compared to S at 12 hai (720 upregulated and 487 downregulated), followed by the PR compared to S at 12 hai (429 upregulated and 293 downregulated) (**Figure 1B**). At 12 hai, *E. pisi* triggered large transcriptional changes across all accessions, but by 48 hai, the number of DEGs had substantially decreased in all comparisons. The number of common DEGs across all accessions remained relatively stable over time (164, 181, and 170 for 0, 12, and 48 hai, respectively). However, at 72 hai, only 93 common DEGs were detected (**Figure 1F**). Notably, transcriptional changes at 12 and 72 hai progressively increased from PS to PR and R accessions (**Figures 1D, F**).

For RNA-Seq validation we observed a high Pearson’s correlation (r=0.98) between the log fold change (logFC) values obtained by RNA-Seq and RT-qPCR for the nine genes tested. Additionally, linear regressions were fitted showing a coefficient of determination (R^2^) of 0.97 (**Supplementary Figure S4A**). Looking at the comparative heat map with both RT-qPCR and RNA-Seq data most expression patterns seemed analogous between RT-qPCR and RNA-Seq throughout the *E. pisi* infection in all accessions (**Supplementary Figure S4B**), technically validating our RNA-Seq datasets.

### 3.2 Constitutive transcriptional differences among *Lathyrus sativus* accessions

At non-inoculated conditions (0 hai), the PR is the accession with the most distinct *E. pisi* defense-related transcriptome compared to the S accession, with 475 upregulated and 347 downregulated DEGs (**Figures 1B, C**). Notably, 321 DEGs were exclusive to the PR compared to S (188 upregulated, 133 downregulated) (**Figure 1C**; **Supplementary Table S2**).

Enrichment analysis revealed that PR-exclusive upregulated biological processes (BPs) included regulation of defense response (GO:0031347), response to jasmonic acid (GO:0009753), and defense response to other organisms (GO:0051707), among other defense-related processes (**Supplementary Table S4**). In these BPs, defense-related DEGs with high logFC included *disease resistance protein RUN1* (g29548.t1, logFC=5.6) and two *Bowman-Birk type proteinase inhibitors* (g11928.t1 and g11927.t1, logFC=5.0 and 3.5).

In the R accession, unique and upregulated DEGs showed enrichment in the phenylpropanoid and lignin biosynthetic processes (GO:0009809, GO:0009699). In contrast, the PS accession displayed a distinct constitutive response compared to R and PR accessions, with upregulated DEGs enriched in BPs related to general cellular and organismal responses to environmental stimuli. This included response to stress (GO:0006950), and processes related to nitrogen metabolism, such as nitrate assimilation (GO:0042128) and the nitrogen cycle (GO:0071941) (**Supplementary Table S4**).

At 0 hai, a total of 164 DEGs were commonly identified among the PS, PR, and R. These included 98 and 60 common upregulated and downregulated DEGs, respectively, and 6 DEGs showing opposite expression, being up or downregulated depending on the accession (**Figure 1C**).

### 3.3 Common and specific *Lathyrus sativus* transcriptional responses against *Erysiphe pisi*

Common DEGs among R, PR, and PS compared to S were predominantly upregulated in all inoculated time points, with enrichment in BPs related to stress and/or stimulus response (GO:0006950, GO:0050896). Upregulated defense-related DEGs included: *probable mannitol dehydrogenase*, (g8997.t1, g8994.t1, g8995.t1 logFC 5.4-8.2); BURP domain protein *RD22* (g22981.t1, g22990.t1, g22980.t1, g22989.t1, g22988.t1, g22982.t1, logFC1.1-4.9); *peptidyl-prolyl cis-trans isomerase FKBP62* and *FKBP65* (g22345.t1, g13946.t1, g28159.t1, logFC 1.0-6.0); *Bowman-Birk type proteinase inhibitor* (g11928.t1, g11927.t1, logFC 1.7-5.0); and *polygalacturonase inhibitors* (g10621.t1, g11046.t1, g28453.t1, logFC 1.0-6.2). Common downregulated BPs across accessions were identified only at 12 hai and relate to starch (GO:0005982, GO:0019252) and glucan (GO:0009250) biosynthetic processes (**Supplementary Table S4**). Overall, the number of downregulated defense-related DEGs was significantly lower compared to the upregulated ones.

Accession-specific DEGs revealed different response strategies among accessions. In the R accession, exclusive upregulated BPs at 12 hai were predominantly linked to physical and chemical defense barriers against pathogen infection. These included cell wall organization and biogenesis (GO:0071555, GO:0071554) and lignin biosynthetic process (GO:0009809). Cell wall organization and biogenesis processes (GO:0071555, GO:0071554) continued to be enriched at 48 hai in the R accession (**Supplementary Table S4**).

At later infection stages (72 hai), the R accession showed upregulated BPs associated with protein folding (GO:0006457), response to osmotic stress (GO:0006970), oxidative stress and detoxification (GO:0006979, GO:0098754), and abscisic acid (ABA) response (GO:0009737). DEGs with putative antifungal functions were exclusively upregulated in R at 12 and/or 72 hai, including *eugenol synthase 1* (g18115.t1, logFC > 3.0) and *thaumatin-like protein 1* (g29711.t1, logFC = 2.9). Additionally, *hypersensitive-induced response protein 1* (g2782.t1, logFC > 1.4) was exclusively upregulated in R across all time points, including non-inoculated conditions (**Supplementary Tables S2**, **S4**).

The BPs previously identified for exclusively upregulated DEGs in PR compared to S at non-inoculated conditions- 0 hai (such as the BP related to response to biotic stimulus) were also observed at 12 hai, with some genes showing increased expression at this later time point (**Supplementary Table S4**). However, at 48 and 72 hai, no specific GO term enrichment was identified for PR. Nevertheless, some defense-related DEGs, such as *Kunitz-type trypsin inhibitor-like 2* and *5* (g3757.t1, g3907.t1) were detected in these time points (**Supplementary Table S2**).

At 12 hai, PR DEGs showed significant enrichment in biotic stimuli, defense and stress BPs. Exclusively downregulated DEGs in PR accession identified at 48 and 72 hai were associated with glycerol transmembrane transport (GO:0015793), polyol transmembrane transport (GO:0015791), carbohydrate catabolic process (GO:0016052), and starch metabolic and biosynthetic processes (GO:0005982, GO:0019252) (**Supplementary Table S4**).

A larger number of DEGs were common between R and PR than between R and PS or PR and PS except at 72 hai, where PR and PS shared more DEGs (132) than R and PR (119) (**Figures 1C–F**; **Supplementary Table S2**). In general, R and PR shared upregulated DEGs related to plant immunity, such as *receptor-like protein Cf-9 homologue* (g8326); cell wall organization, *including 4-coumarate-CoA ligase CCL1* (g17573); antifungal activity, like *Kunitz-type trypsin inhibitor-like 2* and *5* (g3757.t1, g3907.t1); and secondary metabolism genes, including *benzyl alcohol O-benzoyltransferases* (g9735, g9738). R and PR accessions also shared genes involved in jasmonic acid signaling, such as the *allene oxide cyclase* (g13281.t1, g13279.t1, g979.t1). At 12 hai (and at 0 hai), R and PR accessions shared upregulated DEGs enriched in trichome morphogenesis and differentiation (GO:0010026, GO:0010090), as well as plant epidermis morphogenesis (GO:0090626), including DEGs such as *CPR-5* (g20886.t1) and *SCAR2* (g12176.t1 and g12209.t1). At 72 hai, these DEGs were also shared between R and PS. At 48 hai, both resistant accessions (R and PR) had upregulated DEGs involved in responses to abiotic stresses, including reactions to inorganic substances, chemicals, oxygen-containing compounds, and general environmental stressors. Additionally, at both 48 and 72 hai, R and PR shared upregulated DEGs related to water stress responses, such as *NAC domain-containing protein JA2L* (g14943.t1 and g1304.t1).

Regarding the exclusive DEGs in PS compared to S at 12 and 48 hai, the upregulated group was predominantly associated with responses to environmental stimuli. Additionally, we found upregulated DEG terms associated with defense mechanisms and responses to external biotic interactions both in PS compared to S only at 48 hai. At 72 hai, no BPs were enriched for upregulated DEGs (**Supplementary Table S2**).

### 3.4 Temporal gene expression dynamics in *Lathyrus sativus* response to *Erysiphe pisi*

**Figure 2:**
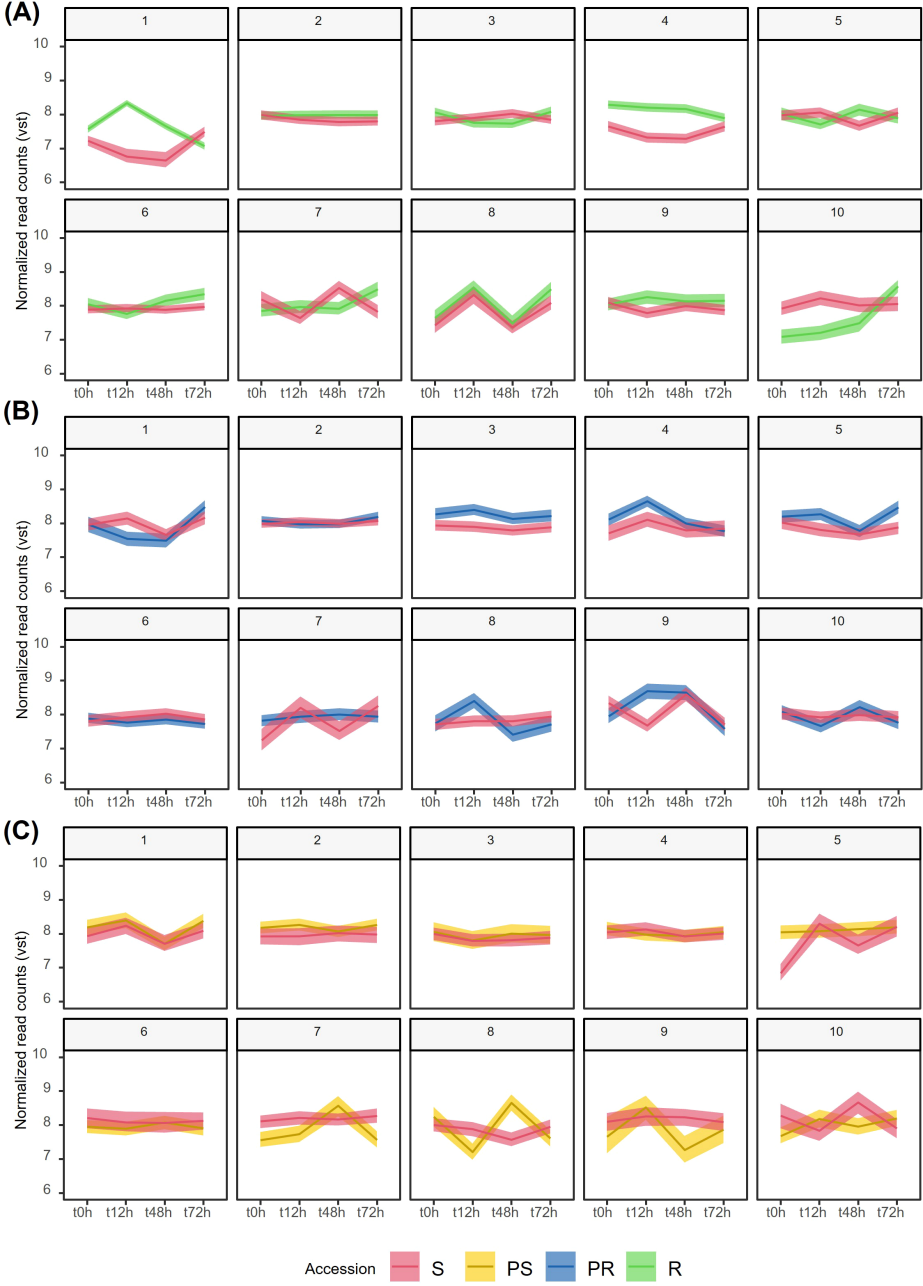
Grouping of temporal gene expression dynamics of *Lathyrus sativus* after *Erysiphe pisi* infection by K-means clustering of vst-normalized DEGs for each accession. For the specific DEGs identified in R, PR, and PS accessions, we plotted the corresponding expression patterns in S. **(A)** R and S, **(B)** PR and S, **(C)** PS and S. R, resistant; PR, partially resistant; PS, partially susceptible; S, susceptible.

From the three independent K-means clustering analyses (**Figure 2**; **Supplementary Table S5**), we detected specific DEG clusters exhibiting a clear differential pattern along infection (**Figure 2**). Regarding the R accession, it was notable that clusters 7, 9, and 10 contained consistently upregulated DEGs in inoculated conditions compared to 0 hai (**Figure 2C**). Common BPs found in these three clusters included response to chemicals, stimulus, and stress. In cluster 7, DEGs were mainly involved in response to oxidative stress (GO:0006979, GO:0098754, GO:0042744), such as five *peroxidase 4* genes (g20878.t2, g20880.t1, g1197.t1, g8235.t1, g1192.t1). *Peroxidase 4* DEGs were also upregulated in R compared to S, especially at 72 hai (**Supplementary Table S2**). Cluster 10 contained DEGs that showed the most pronounced upregulation after inoculation, particularly at 72 hai (**Figure 2C**). In this cluster, DEGs were enriched for GO terms related to cellular responses to changes in oxygen levels (GO:0001666, GO:0036293, GO:0070482, GO:0071456, GO:0036294, GO:0071453, GO:1901700), to heat and temperature stimuli (GO:0009408, GO:0009266), as well as general stress responses such as protein folding (GO:0006457). Notable examples include class II *heat shock proteins* that respond to both temperature and oxygen level changes (g21471.t1, g21472.t1, g14212.t1, g22760.t1, g22740.t1), as well as other *heat shock proteins* that are generally upregulated in R compared to S (**Supplementary Tables S2**, **S6**). Notably, in cluster 10, DEGs were more highly expressed in the S accession at 0, 12, and 48 hai compared to R, indicating that BPs related to cellular responses to changes in oxygen levels, heat and temperature stimuli, and protein folding (**Supplementary Table S6**) were more important for resistance to *E. pisi* at 72 hai (**Supplementary Table S2**).

The expression dynamics of R DEGs in clusters 1 and 6 showed distinct patterns: in cluster 1, DEGs were highly expressed during the early response to *E. pisi* infection (12 hai), with expression levels decreasing at later time points (48 and 72 hai). In contrast, cluster 6 exhibited the opposite trend, where DEGs had a lower expression at 12 hai but an increase in expression at 48 and 72 hai (**Figure 2C**). DEGs more expressed at 12 hai (cluster 1) are mainly involved in DNA replication, confirmation and unwinding (GO:0006268, GO:0071103, GO:0006270, GO:0032392, GO:0032508), chromosome organization (GO:0051276), cutin and cuticle biosynthesis/development (GO:0160062, GO:0010143), and cell wall organization or biogenesis (GO:0071554, GO:0071555). Conversely, the BPs that were more prominently regulated during the later stages of infection (cluster 6) included the response to hormones (GO:0009725) and hormone-mediated signaling pathways (GO:0009755), in addition to the general response to stress and stimuli. Examples of DEGs enriched in these BPs are: *ethylene-responsive transcription factors* (g1394.t1, g6994.t1, g8619.t1, g19521.t1, g22398.t1), *NAC domain-containing proteins 21/22* (g1645.t1), *peptidyl-prolyl cis-trans isomerase CYP40* (g28974.t1). (**Supplementary Tables S2**, **S6**).

In the PR accession, clusters 3, 4, and 8 contained DEGs that may play crucial roles in early response stages (12 hai) but decrease their expression at 48 and 72 hai, even when compared to 0 hai (**Figure 2B**). Many of the DEGs in clusters 3 and 8 were functionally enriched for response to other organisms (GO:0051707, GO:0044419), and response to biotic stimulus (GO:0009607, GO:0043207). This is also the case of cluster 9, where DEGs involved in these BPs were also upregulated at 48 hai (**Figure 2B**). Most of the BPs enriched in cluster 4 were shared with clusters 3, 8, and 9, covering responses to stress (GO:0006950), response to stimulus (GO:0050896), response to chemicals (GO:0042221), and response to abiotic stimulus (GO:0009628). Cluster 6 included DEGs that exhibited reduced expression at all inoculated time points compared to the non-inoculated condition and to the expression profile in S (**Figure 2B**). These DEGs were enriched in general GO terms related to the response to stimulus (GO:0050896, GO:0006950), and more specifically in negative regulation of SAR (GO:0010113).

In the PS accession, clusters 4 and 5 were primarily associated with general stress and stimulus responses, with expression levels similar between non-inoculated and inoculated conditions (**Figure 2A**). Conversely, DEGs enriched for stimulus and hormone responses in cluster 8 expressed less at 12 and 72 hai than at 0 and 48 hai (**Figure 2A**). These include DEGs such as *ethylene-responsive transcription factors* (ERF23/34, g31009.t1, g13966.t1). Lastly, in cluster 10, BPs related to secondary metabolism, including the phenylpropanoid biosynthetic process (GO:0009699), were upregulated following inoculation, particularly at 12 and 72 hai. (**Supplementary Tables S2**, **S6**). Overall, the S accession exhibited more similar and overlapping patterns with PS (clusters 1, 2, 3, 4, and 6) than with PR (clusters 2 and 6) or R (clusters 2 and 8) accessions, patterns that were overall stable from 0 to 72 hai, except for cluster 1 in PS and cluster 7 in R (**Figures 2A–C**, red lines).

To further investigate the gene expression dynamics during *E. pisi* infection, we quantified by RT-qPCR the expression of selected defense-related DEGs in two additional time points: 6 hai and 24 hai (**Supplementary Figure S5**). The expression of *FKBP65* (upregulated in R, PR, and PS compared to S, was consistent across all accessions, with higher expression at 12 hai, followed by a decrease in expression reaching lower expression levels at 24 or 48 hai and then a secondary increase at 72 hai especially in R where expression spiked at 72 hai (**Supplementary Figure S5**).

The *Kunitz trypsin inhibitor 5* was upregulated in R and PR compared to S (**Supplementary Table S2**) and displayed similar expression trends between S and R accessions, with S showing lower expression levels. PS and PR accessions also shared similar expression profiles. Notably, at 72 hai, expression levels decreased in S and PR but increased in PS and R (**Supplementary Figure S5**).

*NAC domain-containing protein JA2L* (upregulated at 48 and 72 hai in R and PR compared to S) was barely expressed in S and PS accessions but showed a consistently increasing expression in R across all time points (**Supplementary Figure S5**). *Eugenol synthase 1* was not expressed in the PS accession but followed the same expression pattern in R, S, and PR, with higher expression at 12 and 72 hai. PR showed a notably higher expression level compared to R and S at 72 hai. R was the only accession with *eugenol synthase 1* early expression at 6 hai (**Supplementary Figure S5**).

### 3.5 Expression of *Lathyrus sativus* NLR genes during *Erysiphe pisi* infection

Among the 3,109 DEGs detected when comparing the R, PR and PS accessions to the S within each time point, we identified a total of 52 NLR genes: 22 CC-NLRs, 25 TIR-NLRs, 3 CC_G10_-NLRs, and 2 CC_R_-NLRs (**Supplementary Table S2**).

**Figure 3:**
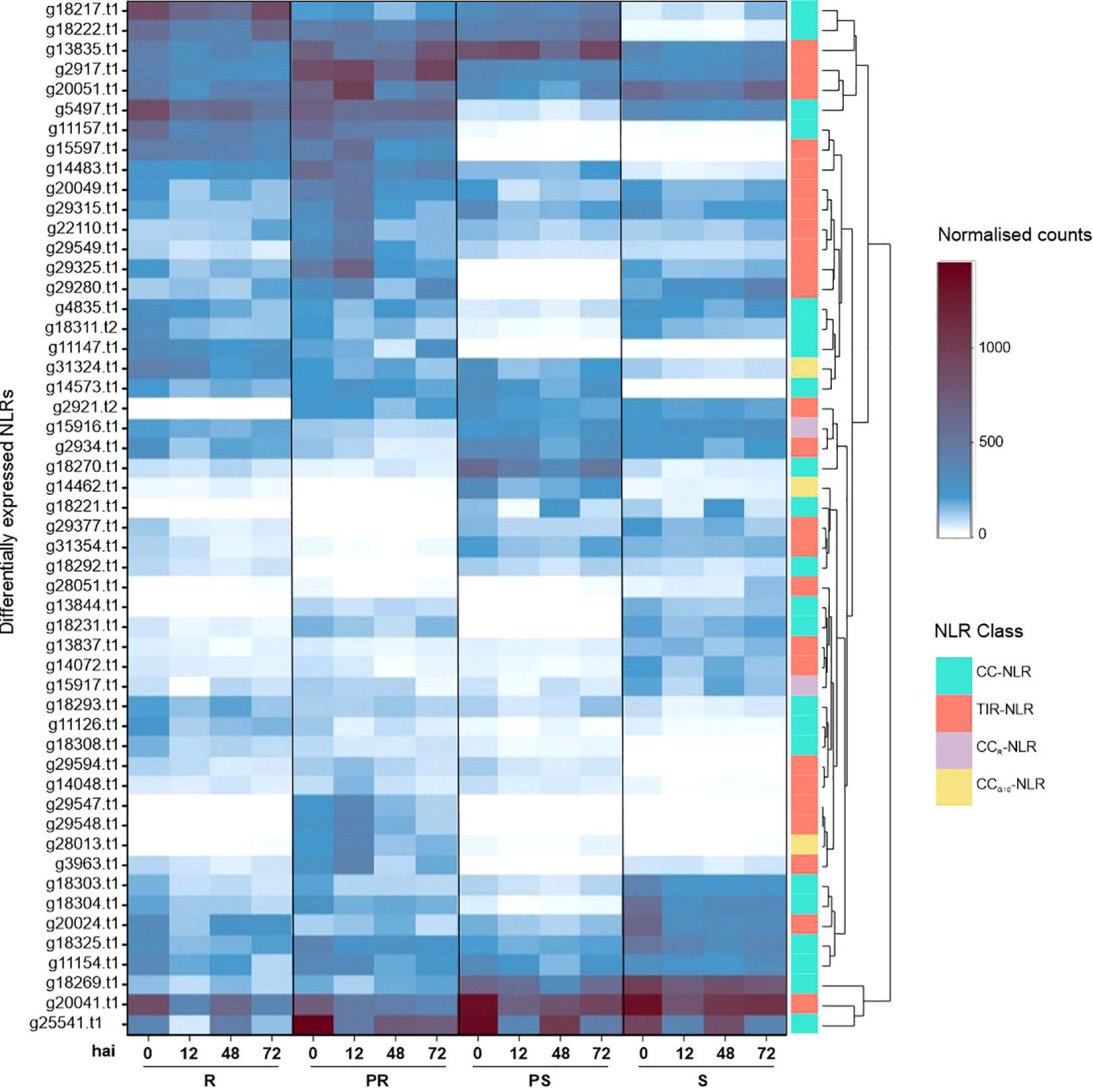
Expression heat map of differentially expressed NLRs in four contrasting *Lathyrus sativus* accessions at 0, 12, 48 and 72 hours after *Erysiphe pisi* inoculation. NLR classes are represented in the last column with the following color code: CC-NLR, turquoise; TIR-NLR, salmon; CC_R_-NLR, lilac; CC_G10_-NLR - yellow. K-means clustering clustered NLR expression patterns. R, resistant; PR, partially resistant; PS, partially susceptible; S, susceptible.

Analyzing the normalized read counts of each identified NLR allowed to investigate expression intensity across the four accessions and time points. In total, sixteen NLRs were highly expressed in all accessions, with at least 100 average normalized counts (shown by light blue) in one of the time points (**Figure 3**). These included six *RUN1* homologues (g2917.t1, g13835.t1, g20024.t1, g20051.t1, g22110.t1, and g29549.t1), three *RPP13-like proteins* 1 (g18269.t1, g18303.t1, g18325.t1), *RGA2* (g11154.t1), *SUMM2* (g31324.t1) and a homologue of the *At5g66900* gene also known as N-requirement gene 1 (*NRG1.1*) (g15916.t1). On the other hand, 36 NLRs (69%) were accession-specific, meaning they were absent or lowly expressed (<100 counts) in at least one accession (**Figure 3**). The time point with the highest number of upregulated differentially expressed NLRs was 12 hai (23) (**Figure 3**; **Supplementary Table S2**). In contrast, 72 hai was the time point with more downregulated NLRs (21) (**Figure 3**; **Supplementary Table S2**). At 12 hai, the PR accession showed the largest amount of upregulated NLRs (18), followed by PR at 0 hai (15), R at 0 hai (12), PR at 72 hai (11), and R at both 12 and 48 hai (10) (**Supplementary Table S2**; **Figure 3**).

Most differentially expressed NLRs (39/52) belonged to the CC- and TIR-NLR classes: *RUN1* (18), *RPP13-like proteins 1* (12), *RGA* genes (5) and *RPM1* (4). *RUN1* TIR-NLRs were found upregulated in all accessions and time points, especially in PR and 12 hai samples (**Figure 3**; **Supplementary Table S2**). *RUN1* g15597.t1 was highly upregulated for R and PR accessions (**Figure 3**). *RPP13-like proteins 1* CC-NLRs were also differentially expressed throughout accessions and time points, with g14573.t1 having notably high logFC in all accessions (**Supplementary Table S2**). *RGA1* CC-NLR (g11157.t1) was highly expressed in R and PR accessions, across all time points (**Figure 3**). *RGA2* (g11154.t1) was only downregulated for R at 72 hai (**Supplementary Table S2**). *RGA3* homologue g11147.t1 behaved similarly to *RGA1*, but g11126.t1 upregulation was R-specific at 0 and 72 hai (**Supplementary Table S2**). *RPM1* CC-NLR g18222.t1 was highly upregulated in all samples, while g18217.t1 was upregulated in all R and PS samples (**Supplementary Table S2**). Two *RPM1* homologues were downregulated: g18221.t1 in PR and R, g18231.t1 for all PS samples and R at 72 hai (**Supplementary Table S2**). Additionally, we identified *SUMM2* (g31324.t1), upregulated at 0, 12 and 48 hai in R samples; *At4g27190* (g14462.t1) upregulated at 0, 48 and 72 hai samples in the PS accession; two *NRG1.1* homologues downregulated, g15916.t1 at 72 hai in the PR accession, and g15917.t1 at 12 hai in the R accession.

### 3.6 *Erysiphe pisi* effector candidates exhibit infection-stage specific expression patterns

To predict effectors from the Ep-CO-01 *E. pisi* isolate, we assembled a *de novo* transcriptome, implementing a combined approach that integrated the identification of effector-like motifs with protein structural comparisons to well-characterized effector proteins. From 297 potential effector candidates, we were able to obtain 163 (**Supplementary Table S7**) high-confidence structural prediction proteins (pTM score>0.5). From these, 40 showed structural similarity to known fungal effectors from the Foldseek database (**Supplementary Table S7**). The most common species to have a structurally similar protein to the *E. pisi* effector candidates were *Puccinia graminis*, *Magnaporthe oryzae, Rhizoctonia solani*, *Phytophthora infestans*, and the cereal powdery mildew causal agent *Blumeria graminis* (**Supplementary Table S8**).

**Figure 4:**
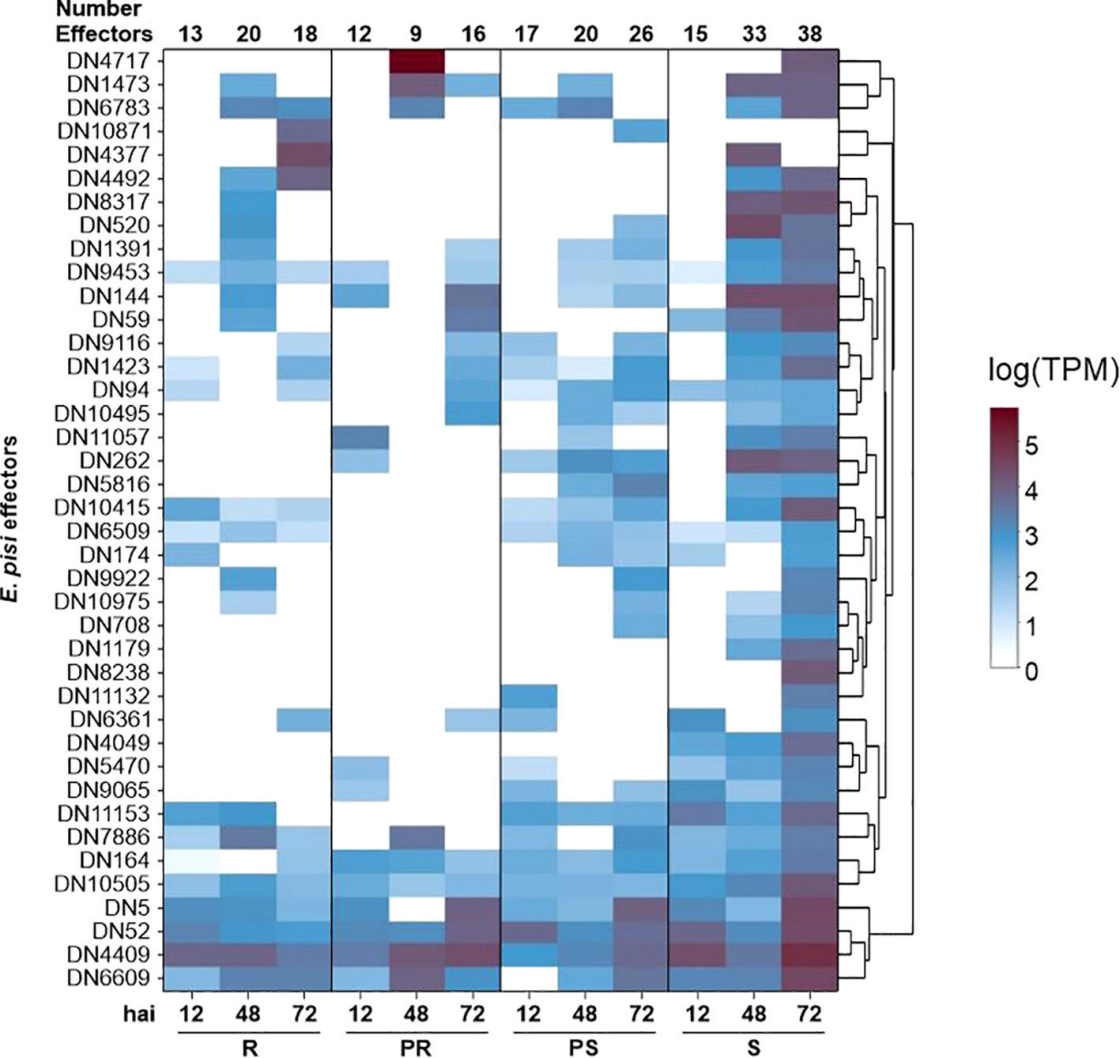
Expression heat map (in transcripts per million) of potential *Erysiphe pisi* effectors at 12, 48 and 72 hours after inoculation (hai) in *Lathyrus sativus* accessions with contrasting powdery mildew responses. R, resistant; PR, partially resistant; PS, partially susceptible; S, susceptible.

Among the 40 effectors identified, the majority belonged to families with broad roles, being categorized as secreted proteins, cell surface proteins, and various enzymes. However, some had sequence similarities to well-studied effectors from other species, such as a homologue of the *Magnaporthe oryzae SnodProt1* (**Supplementary Table S8**). From the 40 *E. pisi* effectors, 35 candidates exhibited sequence similarity to other proteins from the *Erysiphe* genus (**Supplementary Table S8**). By analyzing the read counts of the predicted effectors across the infected samples, we observed that the S and PS accessions showed a consistent increase in the number of expressed effectors over time, rising from 15 to 38 in S, and from 17 to 26 in PS (**Figure 4**). In contrast, R and PR accessions kept the number of expressed effectors lower. In the R accession, 13 effectors were expressed at 12 hai, increasing to 20 at 48 hai, but then decreasing to 18 at 72 hai. In the PR accession, 12 effectors were expressed at 12 hai, 9 effectors expressed at 48 hai, and 16 effectors expressed at 72 hai (**Figure 4**).

We found 16 effector candidates shared among all accessions, including two secreted effector proteins (DN1391, DN144), a 7-dehydrochoslesterol reductase (DN10505), an alpha-glucosidase (DN9453), the SnodProt1 (DN1473), and a subtilisin-like protease 2 (DN7886) (**Figure 4**; **Supplementary Table S7**). We also found a pectin lyase-like protein (DN174) and a secreted glycosidase (DN9922) both expressed in R, PS and S samples at different time points (**Supplementary Table S7**). Three effectors were only present in PS and S: a bacterial alpha-l-rhamnosidase domain protein (DN11132), a serine-threonine protein phosphatase (DN708), and a V-type proton ATPase subunit C (DN5816). We identified three specific effectors in the S accession: a fungal-specific transcription factor domain-containing protein (DN8238), a histone acetyltransferase ELP3 (DN4049), and a pre-rRNA processing protein (DN1179). The number of expressed *E. pisi* effectors was maximum at 72 hai in the S accession, where 38 out of 40 effector candidates were found expressed (**Figure 4**; **Supplementary Table S7**).

## 4 Discussion

In this study, we employed a dual transcriptomics approach to gain deeper insights into the molecular interactions of four *L. sativus* accessions exhibiting contrasting responses to *E. pisi*, during the initial stages of powdery mildew infection. The transcriptional profiles highlighted how diverse *L. sativus* genetic backgrounds modulate their interaction with *E. pisi*, revealing distinct defense mechanisms specific to each accession, while also identifying key common defense responses. Additionally, we analyzed the expression of defense-related NLR genes across all accessions and time points, finding that NLR expression depends on the plant’s genetic background. Among these NLRs, we found differences in NLR genes that could be involved in powdery mildew resistance, such as an *RPP13-like protein 1*, an *RGA1*, two *RPM1* genes, two *RUN1* genes and an *RGA3*. On the pathogen side, we detected putative *E. pisi* effectors that employ different virulence strategies. While some effectors were already described, most belonged to broad and previously undescribed categories in *E. pisi*, further expanding our knowledge on the infection mechanisms of this pathogen.

*Erysiphe pisi* triggered a biphasic *L. sativus* response, characterized by an initial burst in differential gene expression at 12 hai, followed by a quiescent phase at 48 hai, during which the pathogen continued its development but triggered much lower levels of differential gene expression. A second wave of intense gene expression was observed at 72 hai. This trend was consistently observed when additional time points were included for RT-qPCR analysis of selected genes, with expression at 6 hai and 24 hai mirroring that at 12 hai and 48 hai, respectively. A similar biphasic response has also been documented in soybean (*Glycine max*) infected by rust (*Phakopsora pachyrhizi*), suggesting a conserved defense strategy across legume species when facing aerial diseases caused by fungal pathogens which rely on haustoria formation (van de Mortel et al., 2007; Schneider et al., 2011).

On the pathogen side, we noticed that effector expression increased over time, peaking at 72 hai, a trend also observed for the powdery mildew agent *Blumeria graminis f.* sp. *tritici* (Hu et al., 2018). The larger number of effector transcripts at the later time point is likely due to an increased presence of *E. pisi* cells in the samples, although R and PR accessions were more successful in suppressing effector expression over time. Many of these predicted effectors showed protein structures similar to proteins found in other pathogenic fungi such as *Puccinia graminis, Magnaporthe oryzae, Rhizoctonia solani, Phytophthora infestans*, and *Blumeria graminis*.

Most transcriptomic studies on plant host responses to pathogens focus primarily on complete resistance (described as having an incompatible reaction with the pathogen) and complete susceptible accessions, often overlooking the nuances between complete and partial resistance or susceptibility - key factors for developing more durable plant resistance to pathogens. In this study, we selected four *L. sativus* accessions (R, PR, PS, and S) from a worldwide collection previously phenotyped for response against *E. pisi* using detached leaves (Martins et al., 2023). These phenotypes were confirmed in the present study, using whole seedlings, with a progressive increase in macroscopic DS from the R, PR, PS, to the S accession. Although the R accession displayed a compatible reaction with *E. pisi* at 14 dai (IT=3, DS=1.5), our transcriptomic analysis focused on the period from 0 to 72 hai - a period where no visible macroscopic differences were observed among accessions.

*L. sativus* is known for its remarkable resilience to pests and diseases, offering valuable diverse genetic resistance against major fungal diseases in legumes (Vaz Patto et al., 2006; Vaz Patto and Rubiales, 2014a; Almeida et al., 2015). Indeed, even the most susceptible *L. sativus* accession in our study showed lower DS compared to the pea cultivar ‘Messire’, used as a susceptible control, highlighting *L. sativus* generally higher resistance to *E. pisi* compared to pea.

### 4.1 Different *Lathyrus sativus* accessions activate common mechanisms in response to *Erysiphe pisi* inoculation

We observed common defense responses against *E. pisi* infection across PS, PR and R accessions, with enrichment in stress and/or stimulus response processes in all time points after inoculation. This indicates that accessions with contrasting resistance phenotypes activate common mechanisms in response to pathogen-induced stress, though the intensity (fold change) and timing (constitutively, early, or late during infection) of these responses differ. The key common resistance mechanisms in *L. sativus* against *E. pisi* involve antifungal proteins, cell wall reinforcement, ROS-mediated defense, and SAR. At early infection stages (12 hai), starch and glucan biosynthesis processes were downregulated across PS, PR and R accessions, indicating an early metabolic shift from growth-related processes to defense responses under pathogen attack (Chaliha et al., 2018), as also reported in *M. truncatula* infected by *E. pisi* (Gupta et al., 2020).

Examples of defense-related genes common to PS, PR and R accessions included Bowman-Birk type proteinase inhibitors (BBIs), peptidyl-prolyl cis-trans isomerase *FKBP62*, BURP domain proteins (*RD22*), polygalacturonase inhibitors (PGIPs), mannitol dehydrogenases, and flavin-containing monooxygenases. BBIs are well-known for their antifungal properties, blocking proteases secreted by pathogens, preventing the degradation of plant proteins and hindering pathogen growth (Gitlin-Domagalska et al., 2020). BBIs represent a well-conserved defense strategy common in legumes, which seems to be crucial for resisting *E. pisi* infection (Gitlin-Domagalska et al., 2020).

FKBP62 participates in several defense mechanisms, including the accumulation of callose in the cell wall, which reinforces structural barriers against pathogen invasion (Pogorelko et al., 2014; Ge et al., 2022). This mechanism resembles the *er1* (*PsMLO1*) gene function, where the loss of function of the MLO1 protein in pea leads to increased protein cross-linking in host cell walls, creating a physical barrier that prevents penetration by the pathogen (Iglesias-García et al., 2015).

BURP domain proteins (RD22), typically associated with abiotic stress responses, may also play a role in biotic stress through cell wall reinforcement (Yu et al., 2022). Similarly, polygalacturonase-inhibiting proteins (PGIPs) enhance structural defenses by inhibiting fungal polygalacturonases, enzymes that degrade cell wall pectin to allow pathogen entry (Kalunke et al., 2015). Indeed, we detected effectors specialized in cell wall hydrolysis (Lee et al., 2023) such as an alpha-glucosidase (DN9453) expressed in all samples, a hemicellulose-degrading Mannan endo-1,6-alpha mannosidase DCW1 (DN4717) expressed in PR and S accessions, a pectin lyase-like protein (DN174), and a secreted glycosidase (DN9922) both expressed in all accessions but the PR accession. By blocking these enzymes, PGIPs prevent cell wall breakdown, fortifying the plant’s structural defenses against *E. pisi*. In legumes, PGIPs are involved in interactions with apoplastic peroxidases, leading to increased lignin production (Wang et al., 2012). In *L. sativus*, these proteins were upregulated in all accessions compared to the S, with higher expression in the R and PR accessions.

Mannitol dehydrogenases were highly upregulated across all time points in PS, PR and R accessions in response to *E. pisi* infection, especially in the R accession at 48 and 72 hai. Mannitol, a polyol commonly found in fungal spores, fruiting bodies, and mycelia, is secreted by fungi (including Erysiphales) to neutralize reactive oxygen species (ROS), which mediate plant host defenses (Solomon et al., 2007; Meena et al., 2015). To counter this, pathogen-induced plant mannitol dehydrogenases break down fungal mannitol, restoring ROS activity and preventing pathogen spread (Meena et al., 2015). Notably, at 72 hai, several biological processes involved in oxidative stress responses were upregulated across all accessions compared to the S accession.

Flavin-containing monooxygenase 1 (*FMO1*), another key gene upregulated across all comparisons, is a known SAR marker, a long-lasting defense mechanism that is triggered by pathogen-induced cell death and protects the plant from several pathogens beyond the immediate fungal threat (Durrant and Dong, 2004; Mishina and Zeier, 2006; Olszak et al., 2006).

When analyzing the expression patterns of predicted NLRs, *RUN1*, *RPP13-like protein 1*, *RGA2* and *NRG1.1* were particularly highly expressed across all *L. sativus* accessions. Their role in defense against various powdery mildew pathogens is well-documented (Wang et al., 2014; Cheng et al., 2018; Marimon et al., 2020; Bhosle and Makandar, 2021; Massonnet et al., 2022; Zhang et al., 2022), highlighting their potential involvement in *L. sativus*’ response to *E. pisi.* As example, *NRG1.1*, also known as *NRG1A*, is an extensively studied helper CC_R_-NLR characterized by its resistance to powdery mildew 8 (RPW8) domain, which controls resistance to a broad range of powdery mildew pathogens (Xiao, 2004). *NRG1* is required for signal transduction of many sensor TIR-NLRs such as *RUN1*, and can cause autoimmunity when mutated, highlighting its importance in triggering HR (Xiao, 2004; Wu et al., 2019; Sun et al., 2021). Another consistently highly expressed NLR was an *RPS2-like* CC_G10_-NLR (g20538.t1) previously found in linkage disequilibrium with a SNP marker associated with *L. sativus* resistance to *E. pisi* (Martins et al., 2023).

### 4.2 R and PR accessions prioritized physicochemical barriers, secretion of antifungal compounds, and immune signaling pathways

Transcriptome reprogramming in response to *E. pisi* infection revealed many similarities between R and PR accessions, suggesting that both accessions converge on common gene expression patterns to combat *E. pisi* infection. The common defense mechanisms in R and PR accessions involve a multifaceted strategy including physical barriers, chemical defenses, antifungal proteins, ROS quenching, NLR expression, HR, and hormone signaling pathways, with the R accession showing a faster and stronger activation than the PR accession.

The high expression observed for genes related to processes like cell wall reinforcement, phenylpropanoid metabolism, and flavonoid biosynthesis both in R and PR accessions, has also been reported in *L. cicera*, *P. sativum* and *M. truncatula* responses to *E. pisi* (Foster-Hartnett et al., 2007; Santos et al., 2020; Bhosle and Makandar, 2021). One key enzyme in the phenylpropanoid pathway, 4-coumarate-CoA ligase (CCL1), was already upregulated before inoculation (at 0 hai), increasing its expression at 12, and 72 hai, in both PR and R accessions. *CCL1* is involved in lignin and flavonoid biosynthesis (Cao et al., 2020; Li S. et al., 2020). These compounds play important roles in plant defense with lignin acting as a physical barrier to pathogen penetration (Lee et al., 2019; Saberi Riseh et al., 2024), and with flavonoids providing chemical defense through antifungal activity, ROS quenching, chelation of metal pathogen enzyme cofactors, and triggering HR (Mierziak et al., 2014). Therefore, the elevated expression of *CCL1* in PR and R accessions likely contributes to chemical and physical defenses against *E. pisi* infection.

The expression of genes involved in trichome morphogenesis and plant epidermis development such as *CPR-5* and *SCAR2* may play a role in the R and PR accessions resistance to *E. pisi* constitutively (0 hai) and in the early stages of infection (12 hai). *CPR-5* controls trichome cell cycle transition and activates plant effector-triggered cell death (Peng et al., 2020). *SCAR2* is required for epidermal morphogenesis and regulates trichome branching (Basu et al., 2005; Zhang et al., 2005). Trichomes act as dynamic passive barriers, preventing spores and other microbial elements from reaching the leaf surface (Karabourniotis et al., 2020). Beyond their structural role, trichomes contain phenolic compounds, including flavonoids, which further enhance the plant’s defense. Studies in Cucurbitaceae have shown that increased trichome density and polyphenol accumulation in the epidermis are associated with reduced susceptibility to stem blight (*Didymella bryoniae*) (Rennberger et al., 2017). Similarly, resistance to rust (*Puccinia helianthi*) in sunflower is associated with coumarin and other phenolic compounds’ accumulation on the leaf surface, impairing germ tube growth (Prats et al., 2007a).

In addition to physical or physicochemical barriers, both R and PR accessions showed high expression of genes encoding secreted antifungal proteins like Kunitz-type trypsin inhibitors (KTIs) (Huang et al., 2010; Cai et al., 2018). In *L. cicera*, a KTI was identified as a candidate gene for resistance against *E. pisi* (Santos et al., 2020), supporting their importance in *Lathyrus* species’ defense strategies against this pathogen.

A shared set of DEGs involved in plant immunity was upregulated in both R and PR accessions. An interesting example is the receptor-like protein Cf-9. This receptor-like protein (RLP) has extracytoplasmic leucine-rich repeats (eLRRs) that confer disease resistance through the recognition of fungal effectors, resulting in HR activation (van der Hoorn et al., 2005; Jamieson et al., 2018). Therefore, though structurally similar to a PRR, RLP Cf-9 behaves as an NLR (Jamieson et al., 2018). RLP Cf-9 was highly expressed in R and PR accessions, especially at 0 and 12 hai, suggesting a fast *E. pisi* recognition, immune signaling and prevention of pathogen progression in these accessions. Although HR has not been observed macroscopically in the studied *L. sativus* accessions infected by *E. pisi*, HR is an effective mechanism against biotrophic pathogens, including legume powdery mildew, as observed macro- and microscopically for *Pisum* species (Fondevilla et al., 2006, 2007; Fondevilla and Rubiales, 2012) and *M. truncatula* (Prats et al., 2007b). Therefore, detailed histological studies are required to investigate the role of HR in the *L. sativus* response against *E. pisi*.

Regarding NLR genes potentially involved in the R and PR ETI response, *RUN1* (g15597.t1) and *RGA1* (g11157.t1) showed significantly high expression levels. Notably, *RGA1* was found to be upregulated in other plant-aerial-pathogen interactions, such as *Oryza sativa* infected by *Rhizoctonia solani* (sheath blight) and *Triticum aestivum* infected by *Puccinia striiformis* f. sp. *tritici* (rust) (Zheng et al., 2020; Durgadevi et al., 2021).

Both R and PR accessions upregulated genes involved in the JA signaling pathway, a stress-responsive hormone produced during pathogen attacks that activates key defense mechanisms (Yang et al., 2019). For example, the allene oxide cyclase (AOC), a key enzyme in the biosynthesis of JA, was upregulated in both accessions at all time points. Notably, recent studies on *Medicago* spp. identified *AOC* as pathogen-responsive (Yang et al., 2023).

At 48 hai, R and PR accessions also shared upregulated genes previously linked to abiotic stress responses, particularly water-related stress, which persisted into 72 hai. An example was the *NAC domain-containing protein JA2L*, that was minimally expressed in the PS accession but exhibited significantly higher expression in R and PR accessions at all time points, including at 6 and 24 hai. In particular, the R accession displayed a steady increase in *JA2L* expression from 0 to 72 hai. In tomato, *JA2L* targets genes involved in SA metabolism, a key component of plant defense responses (Kotera et al., 2023). In parallel, SA biosynthesis in R and PR accessions was reinforced through the upregulation of benzyl alcohol O-benzoyltransferases, which, in conjugation with peroxisomal β-oxidative pathway, contribute to pathogen signal-induced SA production (Kotera et al., 2023). In our study, those genes were highly upregulated at 12, 48, and 72 hai in R and PR, highlighting their role in strengthening immune responses against pathogen progression.

### 4.3 The R accession showed a robust constitutive physicochemical defense response

In addition to the common genes and defense mechanisms between R and PR accessions, each of these accessions displayed specific molecular responses to *E. pisi*, contributing to their varying DS values. While physical and chemical barrier reinforcement was common between the accessions, variations in the type, number of DEGs, timing, and intensity (fold change) distinguished their response.

In the R accession, it was clear that the defense strategy combines early and rapid reinforcement of structural barriers with sustained chemical defenses and stress responses. Before inoculation, upregulated BPs specifically found in the R accession were related to lignin biosynthesis and the phenylpropanoid pathway, reinforcing its structural barriers against possible pathogen attack. Additionally, the *hypersensitive-induced response protein 1* gene involved in HR activation (Zhou et al., 2010) was only upregulated in the R accession, not only prior to inoculation, but also across all infected time points, suggesting a role of HR in R defense. Moreover, the CC_G10_-NLR *SUMM2* (g31324.t1) was upregulated at 0, 12 and 48 hai in R accession. *SUMM2* does not directly sense the pathogen effectors; instead, it monitors the phosphorylation status of the plant calmodulin-binding receptor-like cytoplasmic kinase 3, transducing the signal to HR, and can trigger autoimmunity in specific knockout backgrounds (Zhang et al., 2017). This potent autoimmune ability to induce HR on its own is characteristic of CC_G10_-NLRs, also known as the autonomous NLR clade (Lee et al., 2021). Although no macroscopic HR was observed in this study, consistent with previous phenotypic analyzes of *Lathyrus* spp. inoculated with *E. pisi* (Vaz Patto et al., 2006; Santos et al., 2020), we cannot rule out HR as an effective defense mechanism against *E. pisi* in *L. sativus*. Therefore, detailed histological studies are necessary to further explore the potential role of HR in the *L. sativus* response to powdery mildew infection.

Proteins involved in antifungal activity such as *eugenol synthase 1* and *thaumatin-like protein 1* are important in the R accession response to *E. pisi*. Eugenol synthase is an enzyme responsible for the biosynthesis of eugenol, a volatile compound with antifungal activity (Anand et al., 2016; Ulanowska and Olas, 2021). Expression analysis of *eugenol synthase 1* revealed that this gene is exclusively expressed in the R accession at 6 hai, while at all other analyzed time points, it is expressed in the R, PR, and S accessions, albeit at lower levels in the S accession. Thaumatin-like proteins (TLPs) are part of the pathogenesis-related protein family and play a crucial role in providing resistance against various fungi, including *E. pisi* and other pathogens that infect legumes (Jayaprakash et al., 2021; Zhou et al., 2023; Feng et al., 2024). Here, TLP1 was significantly upregulated in R accession at 12 hai.

Upon *E. pisi* infection at 12 hai, alongside the continued emphasis on physical and chemical defenses, the R accession specifically showed high expression of genes related to epigenetic regulation, such as DNA replication, conformation, and unwinding. This suggests that DNA repair, transcription activation, and chromatin reorganization may be involved in the plant’s defense response to the pathogen. In the R accession, DNA unwinding mediated by DNA replication licensing factors, particularly mini-chromosome maintenance (MCM) genes, was important at 12 and 72 hai. MCM proteins ensure that genomic DNA is replicated completely and accurately during the S phase of the cell cycle (Tuteja et al., 2011). Since pathogen exposure affects plant growth, it may directly or indirectly affect cell cycle regulation by altering the endoreduplication process. This may result in DNA replication perturbation and cell death (Tuteja et al., 2011). In pea, the PsMCM6 functions as a helicase, aiding in unwinding the secondary structures of mRNA in stress-responsive genes (Tuteja et al., 2011). In Arabidopsis, *MCM7* is expressed during root knot and cyst nematode infections (Huang et al., 2003). Moreover, studies show that the activation of PTI, ETI, and SAR depends on epigenetic regulation of gene expression (Chen et al., 2017). Thus, these mechanisms are likely important for a robust and systemic response to *E. pisi* in the R accession.

At later stages of infection (72 hai), the R accession transitioned to sustaining resistance through mechanisms that include oxidative stress management, osmotic stress responses, and abscisic acid (ABA) signaling. At this time point, genes involved in heat and oxidative stress responses, such as *peroxidase 4*, and *heat shock proteins*, were strongly upregulated. Additionally, protein folding, and detoxification mechanisms became more prominent, indicating that cellular homeostasis under stress is essential for adaptation and survival. Peroxidases, as members of the pathogenesis-related protein family, play a crucial role in maintaining redox homeostasis within plant cells (Sellami et al., 2022; dos Santos and Franco, 2023). Besides their role in cell signaling after infection, peroxidases contribute to plant defense by polymerizing macromolecules that, once deposited on the extracellular surface, promote cell wall strengthening (dos Santos and Franco, 2023). Additionally, peroxidases can catalyze the oxidative degradation of phenolic compounds in the cell regions damaged by pathogens (dos Santos and Franco, 2023). *Peroxidase 52* was reported as showing very high transcript expression in resistant pea genotypes against *E. pisi*, highlighting the potential role of peroxidases in enhancing resistance mechanisms against this pathogen (Bhosle and Makandar, 2021). Many different heat shock proteins (HSPs) and related transcription factors were specifically and highly upregulated in the R compared to S, particularly at 72 hai. HSPs play diverse roles in plants by acting as molecular chaperones, facilitating protein assembly, stabilization, and maturation (ul Haq et al., 2019). Additionally, HSPs enhance membrane stability and help detoxify ROS by positively regulating antioxidant enzyme systems (ul Haq et al., 2019). As a result, they are crucial in both abiotic and biotic stress responses (Park and Seo, 2015; ul Haq et al., 2019). The role of HSPs in the resistance to *E. pisi* was also reported in *L. cicera* and *P. sativum* (Curto et al., 2006; Santos et al., 2020; Bhosle and Makandar, 2021). Interestingly, *E. pisi* also expressed HSPs Rot1 (DN4492) and chaperone J-domain-containing protein (DN8317) to protect itself from plant defenses.

### 4.4 The PR accession responds to *Erysiphe pisi* by activating biotic stress-specific processes from the earliest infection stage

PR-specific molecular responses against *E. pisi* mainly relied on key BPs related to biotic stress defense and signaling, including the expression of NLR genes. Before inoculation, the PR accession exhibited a specific defense-related transcriptome, distinct from both R and PS accessions compared to S. Prominent defense-related genes constitutively upregulated in PR include 15 NLRs, suggesting this accession is primed for an effective response even before pathogen exposure.

At 12 hai, the PR accession continued to exhibit the strongest defense-focused response, marked by increased activity in processes related to biotic stimuli, including fungal infection. This robust response, sustained from 0 to 12 hai, highlights the PR accession’s ability to react quickly to pathogen presence. However, as the infection progresses to 48 and 72 hai, the transcriptomic activity in defense-related processes declines. Histological observations on *L. sativus* PR and S accessions inoculated with *E. pisi* showed at later stages (48-72 hai) a significant reduction in the number of hyphal branches at later stages (48-72 hai) in PR compared to S (Vaz Patto, personal communication), suggesting that the early and defense-focused PR transcriptional activity may contribute to restrict fungal development. Thus, the overall defense response in the PR accession appeared to be front-loaded, reducing intensity as the infection progressed, which might hinder its long-term effectiveness against sustained pathogen pressure, phenotypically distinguishing this accession from R.

Contrary to the previous paradigm, NLRs have recently shown not to always promote complete resistance and can instead be agents of partial resistance. They may confer non-race-specific resistance like Pik, a CC-NLR conferring complete or partial resistance to *Magnaporthe oryzae* in rice (Varden et al., 2019), I2, a CC-NLR conferring resistance to *Fusarium oxysporum f.* sp. *lycopersici* and to *Phytophthora infestans* in tomato (Giannakopoulou et al., 2015), and RGA5, conferring partial resistance to *Blumeria graminis* in wheat (Liu et al., 2024). NLR proteins can also contribute towards partial resistance by misregulation of NLR gene expression, as observed in avocado infected with *Phytophthora cinnamomi* (Fick et al., 2022b). Enough NLR activation needs to occur for a complete resistance response. If there is insufficient expression of the specific NLR, there is poor detection of pathogen presence, leading to lower levels of immune response activation, and thus the plant can acquire a partial resistant phenotype (Fick et al., 2022a).

### 4.5 The PS accession exhibited a delayed and less specific defense response to *Erysiphe pisi*

The PS accession exhibited a constitutive response pattern mainly focused on general environmental stress rather than on pathogen-targeted defense, reflecting a potentially weaker baseline defense system when compared to the PR and R accessions. Defense-related processes and responses to external biotic interactions were more evident at 48 hai, occurring later than in the R and PR accessions. The NLR *At4g27190* (g14462.t1) was upregulated in the PS samples at 0, 48, and 72 hai. Notably, four homologues of *At4g27190* were upregulated in a resistant *Gerbera hybrida* accession compared to a susceptible accession when challenged with the powdery mildew causal agent *Podosphaera xanthii* (syn. *Sphaerotheca fusca*) (Bhattarai et al., 2020).

### 4.6 The *Erysiphe pisi de novo* transcriptome helped effector prediction

Effectors are crucial for powdery mildew virulence, as they interact with host defense-related proteins to weaken host resistance and promote successful fungal colonization (Hu et al., 2018). Despite focusing only on 40 effectors, we identified 297 potential effectors using the *E. pisi* Ep-CO-01 isolate. Bhosle and Makandar (2021) identified 681 effectors in the Ep01 isolate, while Sharma et al. (2019) reported 167 effectors in the Palampur-1 isolate, both of which infect *Pisum sativum*. Differences between the number of effectors may be due to different isolates and methodological approaches. Notably, 27 of the 167 effectors in Sharma et al. (2019)’s study correspond to 23 of our identified effectors.

From the 40 *E. pisi* effectors with Foldseek hits selected in the present study, 16 were expressed in all accessions at least in one inoculated time point. One of them was a 7-dehydrocholesterol reductase (DN10505), an enzyme known to play a significant role in the sterol biosynthesis pathway, which is involved in *Phytophthora capsici* development and zoospore virulence (Wang et al., 2022). Another example is the SnodProt1 (DN1473), a member of the cerato-platanin protein family that is required for the virulence of different pathogens (Jeong et al., 2007; Zhang et al., 2017a; Nasir et al., 2018). This protein can also function as a plant defense elicitor (Zhang et al., 2017a; Nasir et al., 2018). We also detected two other secreted effector proteins (DN1391, DN144) in all accessions, both containing a ribonuclease/ribotoxin domain. Fungal ribotoxins were observed in other *E. pisi* studies (Gupta et al., 2020; Sharma et al., 2019) and can act as elicitors to trigger HR depending on plant genotype (Yin et al., 2022).

We found six specific effectors only expressing in S and PS. Among them, we identified a fungal-specific transcription factor (DN8238) at 72 hai which could be modulating the expression of relevant pathogenicity-related or even host resistance genes; a secreted serine-threonine phosphatase (DN708) at 48 and 72 hai that can alter the activation state of defense host proteins; a V-type proton ATPase (DN5816) at 48 and 72 hai to provide chemical energy for host-pathogen interactions; an histone acetyltransferase ELP3 (DN4049) in all time points for epigenomic gene modulation, and a pre-rRNA processing protein (DN1179) at 48 and 72 hai denoting high translation activity. These effectors contribute towards a broad transcriptional and translational activity but do not point towards any specific pathogenicity strategy. Low specificity is frequently a characteristic of the most abundant effectors, as targeting multiple host targets enhances the likelihood of blanketing the entire plant defense-signaling network, thereby promoting disease (Khan et al., 2018).

The *E. pisi* genome and its effectorome in interaction with *L. sativus* remained largely unexplored. Furthermore, effectors are an ever-changing family, with pronounced differences even among strains (Khan et al., 2018). In the *de novo E. pisi* transcriptome containing 20,608 transcripts, we identified 8,031 genes, which is within the expected range for powdery mildew fungi of 6,046 to 8,470 genes (Zaccaron et al., 2023). On the host side, this is the first transcriptomic study using the recently released high-quality *L. sativus* genome (Vigouroux et al., 2024). The availability of this genome provided a robust reference for mapping transcriptomic data, enabling precise identification and annotation of DEGs. Despite the high overall functional annotation rate, 24% of DEGs were classified as ‘no annotation’, suggesting these may represent *L. sativus*-specific genes, possibly involved in unique defense mechanisms against *E. pisi* that have yet to be described.

## 5 Conclusion

This study provides valuable insights into the complex molecular interactions and defense mechanisms in *L. sativus* against *E. pisi*, underscoring the genetic diversity in pathogen responses across accessions with contrasting resistance levels. By identifying genes and pathways involved in different resistance mechanisms, breeders can employ pyramiding techniques to combine and integrate them into a single genotype to create varieties with broader resistance spectra. Moving forward, future research should focus on the functional validation of the most promising candidate genes, including NLRs, and the assessment of effector functions. We identified NLRs consistently more expressed in resistant accessions, which could be sensing, directly or indirectly, *E. pisi* effectors. NLRs g11157.t1 (*RGA1*), g11147.t1 (*RGA3*), g15597.t1 and g29548.t1 (*RUN1*), g18217.t1 and g18222.t1 (*RPM1*), and g14573.t1 (*RPP13-like protein 1*) could contribute to *E. pisi* resistance, and we recommend their functional validation in future studies. The characterization of *E. pisi* effectors and their interactions with *L. sativus* defense genes will be crucial for understanding the molecular basis of powdery mildew and developing effective resistance breeding strategies and tools, such as marker-assisted selection. The insights gained from *L. sativus* can also be utilized to enhance resistance in other economically important legumes susceptible to *E. pisi*, such as pea. By improving disease resistance in these crops, we can boost legume yields while promoting more sustainable agricultural practices.

## Data Availability

The datasets presented in this study can be found in online repositories. The names of the repository/repositories and accession number(s) can be found below: https://www.ebi.ac.uk/biostudies/, E-MTAB-14647.
