# Supplementary material for: A dual transcriptome analysis reveals accession-specific resistance responses in Lathyrus sativus against Erysiphe pisi
Source: Front Plant Sci. 2025 Mar 5;16:1542926. doi: 10.3389/fpls.2025.1542926 (PMC11921622; doi:10.3389/fpls.2025.1542926)
Supplement: Supplementary file 1 [file DataSheet1.docx]

**
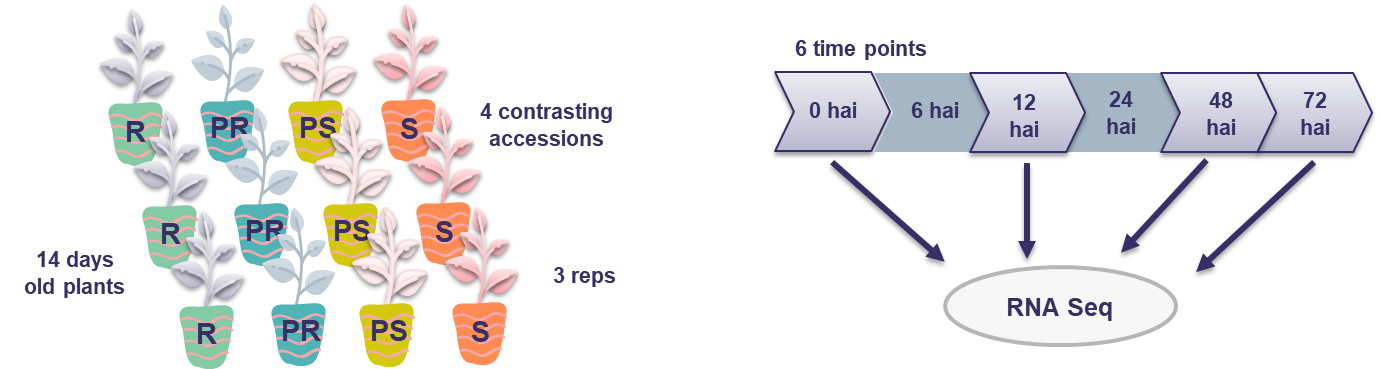
Supplementary Figure 1** Experimental design of the RNA Seq experiment. Three biological replicates of four contrasting *Lathyrus sativus* accessions against *Erysiphe pisi* (S – PI426890, PS – PI426882, PR – PI221467_A, R - PI268478) were inoculated. Infected leaf material from 14 days-old plants was collected at 0, 6, 12, 24, 48, and 72 hai, and the 0, 12, 48, and 72 hai time points were used for RNA Seq.


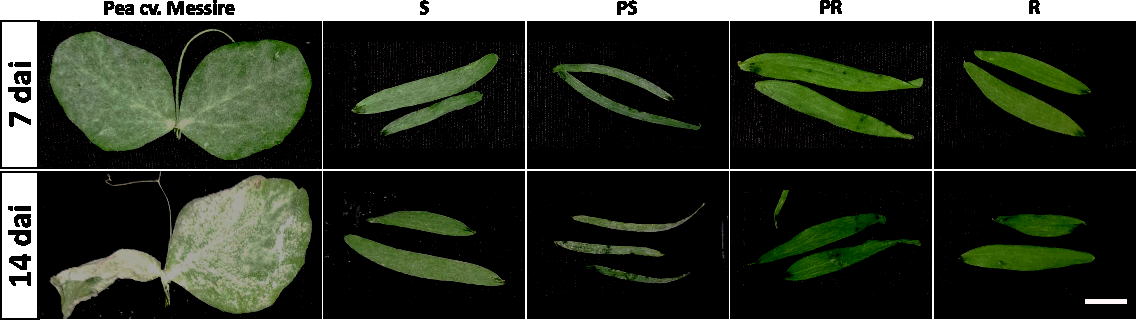


**Supplementary Figure 2** Disease symptoms of the *Lathyrus sativus* accessions PI426890 (S – Susceptible), PI426882 (PS – Partially Susceptible), PI221467_A (PR – Partially Resistant), and PI268478 (R – Resistant) at 7 and 14 days after inoculation (dai) with *Erysiphe pisi*. The pea cv. ‘Messire’ was used as a positive control for inoculation. Scale: 1 cm.

**
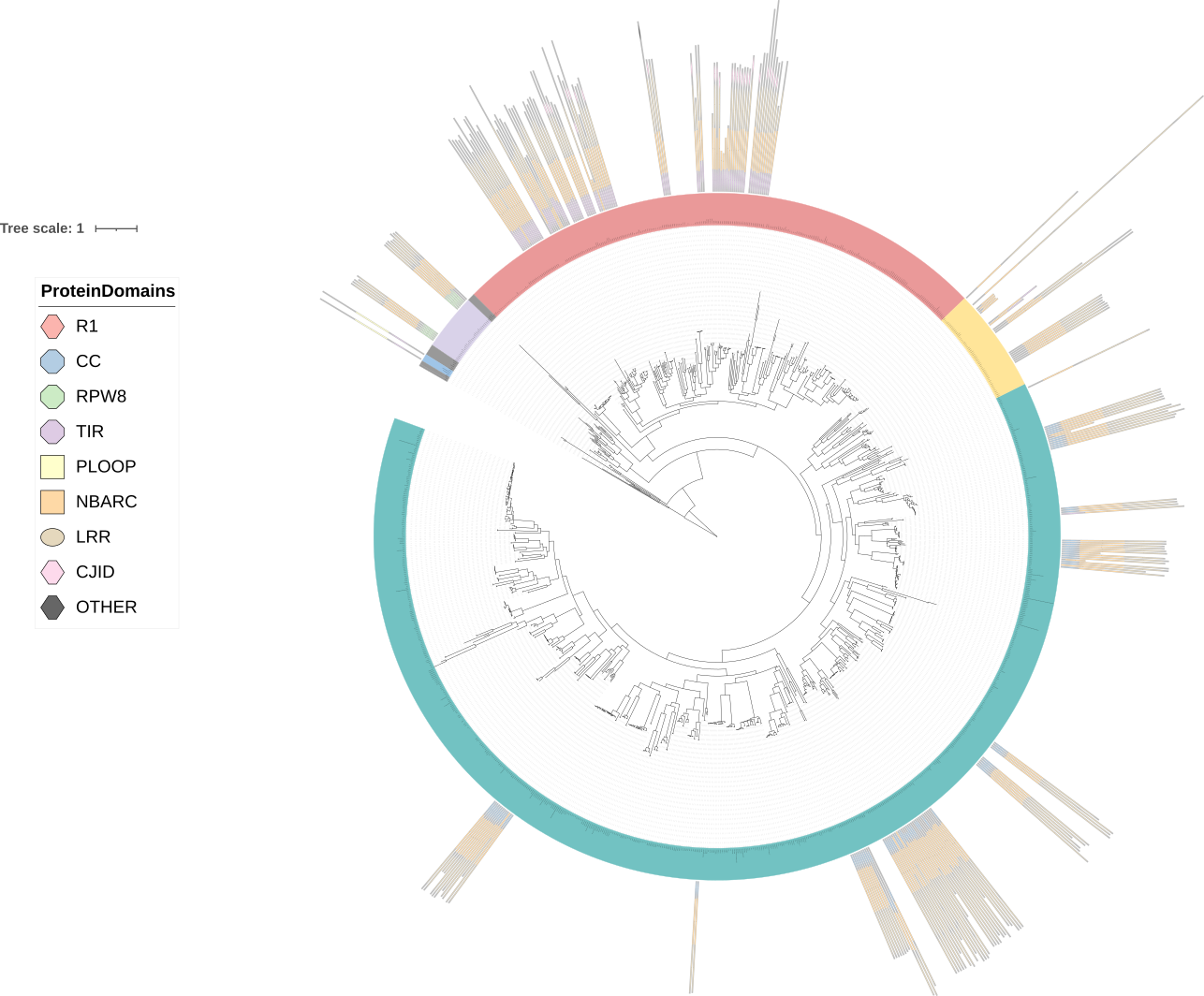
Supplementary Figure 3** *Lathyrus sativus* NLR phylogenetic tree including NLRs from the RefPlantNLR dataset. This image was obtained using the iTOL website. The legend depicts common NLR domains predicted by NLRtracker. NLR classes are represented with the following colour code: CC-NLR – turquoise; TIR-NLR – salmon; CC_G10_-NLR – yellow; CC_R_-NLR – lilac.


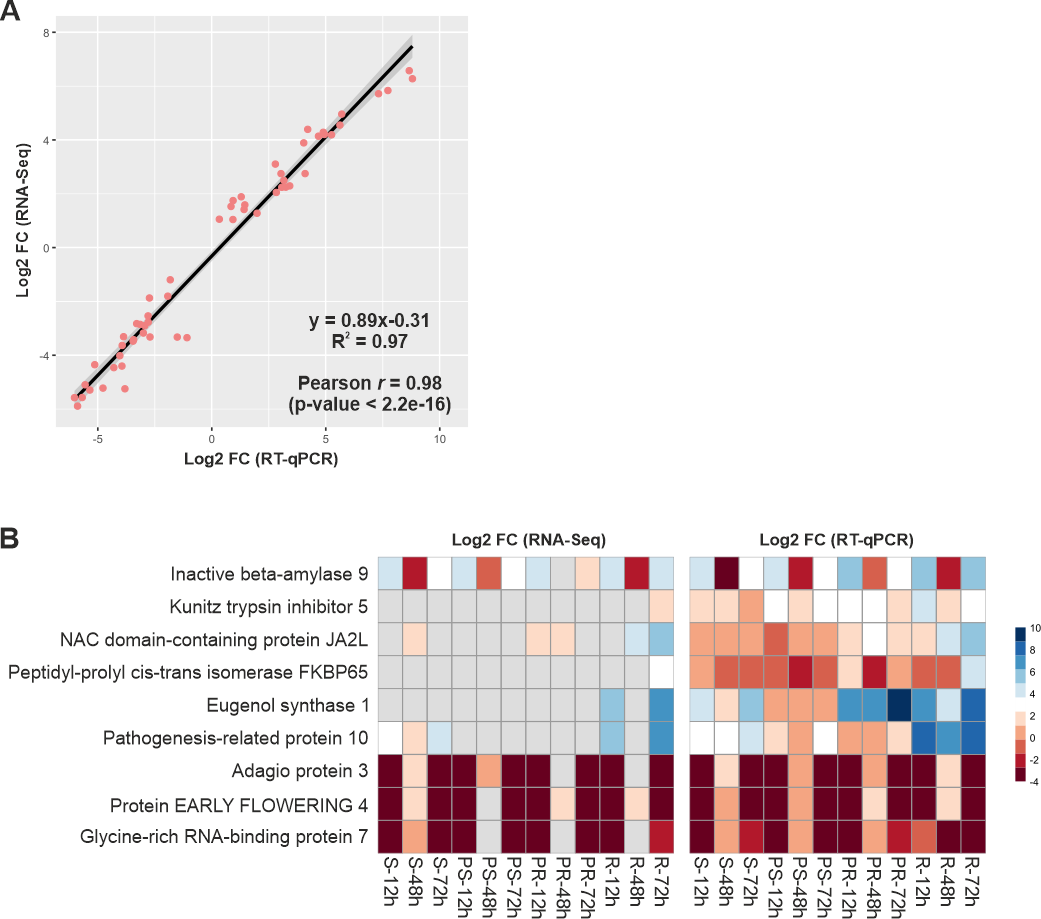


**Supplementary Figure 4** Comparisons of target gene expression by qRT-PCR for technical validation. (A). Linear regression and Pearson correlation analysis between qRT-PCR and RNA-seq results (*r* = 0.933) for the selected genes. X-axis numbers represent the fold change values of qRT-PCR results. Y-axis numbers represent the fold change values of RNA-seq results. (B) Heatmap representing log_2_ fold change expression values from RNA-seq and qRT-PCR for the selected genes.


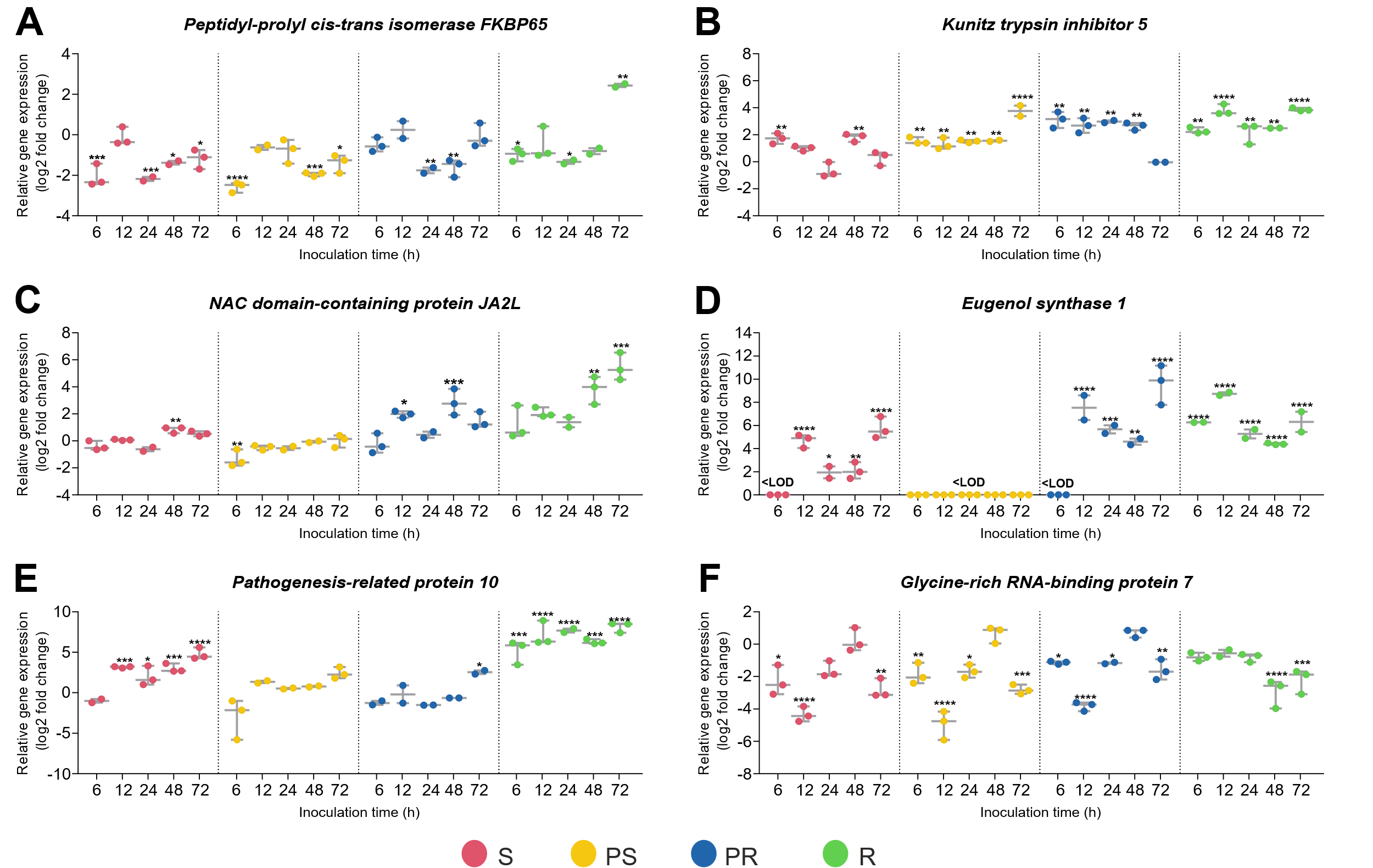


**Supplementary Figure 5**. Gene expression profiles of defence-related DEGs for the S, PS, PR, and R accessions in five time points analysed by RNA-seq (6 hai, 12 hai, 24 hai, 48 hai, and 72 hai) by qRT-qPCR. Results were normalised against the expression levels of *ʎ-tubulin* and *chromodomain helicase DNA-binding protein* using the Pfaffl method (-2ΔΔCt). The log_2_ fold change values were represented in relation to 0 hai (non-inoculated conditions). Individual values (dots) of three independent biological replicates are shown. Red: S – susceptible; Yellow: PS – partially susceptible; Blue: PR – partially resistant; Green: R – resistant accessions.
